# Manual Wheelchair Skills Training for Community-Dwelling Veterans with Spinal Cord Injury: A Randomized Controlled Trial

**DOI:** 10.1371/journal.pone.0168330

**Published:** 2016-12-21

**Authors:** R. Lee Kirby, Doug Mitchell, Sunil Sabharwal, Mark McCranie, Audrey L. Nelson

**Affiliations:** 1 Division of Physical Medicine and Rehabilitation, Department of Medicine, Dalhousie University, Halifax, Nova Scotia, Canada; 2 Charlie Norwood Veterans Administration Medical Center, Augusta, Georgia, United States of America; 3 Veterans Administration Boston Health Care System and Harvard Medical School, Boston, Massachusetts, United States of America; 4 Health Services Research & Development Center of Innovation on Disability and Rehabilitation Research (CINDRR), James A. Haley Veterans’ Hospital, College of Public Health, University of South Florida, Tampa, Florida, United States of America; Case Western Reserve University, UNITED STATES

## Abstract

**Objectives:**

To test the hypotheses that community-dwelling veterans with spinal cord injury (SCI) who receive the Wheelchair Skills Training Program (WSTP) in their own environments significantly improve their manual wheelchair-skills capacity, retain those improvements at one year and improve participation in comparison with an Educational Control (EC) group.

**Methods:**

We carried out a randomized controlled trial, studying 106 veterans with SCI from three Veterans Affairs rehabilitation centers. Each participant received either five one-on-one WSTP or EC sessions 30–45 minutes in duration. The main outcome measures were the total and subtotal percentage capacity scores from the Wheelchair Skills Test 4.1 (WST) and Craig Handicap Assessment and Reporting Technique (CHART) scores.

**Results:**

Participants in the WSTP group improved their total and Advanced-level WST scores by 7.1% and 30.1% relative to baseline (p < 0.001) and retained their scores at one year follow-up. The success rates for individual skills were consistent with the total and subtotal WST scores. The CHART Mobility sub-score improved by 3.2% over baseline (p = 0.021).

**Conclusions:**

Individualized wheelchair skills training in the home environment substantially improves the advanced and total wheelchair skills capacity of experienced community-dwelling veterans with SCI but has only a small impact on participation.

## Introduction

Wheelchairs are among the most important of rehabilitation interventions [1]. Wheelchairs improve mobility and participation, reduce caregiver burden and reduce the likelihood of placement in a long-term-care facility [2–7]. However, there are a number of problems associated with their use. These problems include poor fit [8], frequent need for repairs [9], the role that wheelchairs may play in overuse injuries [10,11] and acute injuries that can occur during use [12,13].

One way to enhance the benefits and minimize the problems of wheelchair use is better wheelchair provision. The World Health Organization (WHO) guidelines on wheelchair provision [14] include 8 steps for the service-delivery process and evidence is accumulating about the positive impact of such a process [15–17]. One of these steps is the training of wheelchair users in the use and care of their wheelchairs.

Despite the well-recognized importance of training, there is a surprisingly low prevalence and/or intensity of wheelchair skills training [18–20]. Earlier studies on community-dwelling people with spinal cord injury (SCI) have found lower than expected skill levels [21–25].

One available resource for addressing this problem is the Wheelchair Skills Training Program (WSTP) [26]. The WSTP is a set of training protocols that combines the best available evidence on motor-skills learning with the best evidence on how to perform specific wheelchair skills. There are a growing number of randomized controlled trials (RCTs) that provide evidence for the safety and effectiveness of the WSTP in a variety of settings [27–32] as well as other evidence about the benefits of wheelchair-skills training [33–35]. There is also growing evidence for a relationship between wheelchair skills and other important outcomes such as confidence and participation [2,3,4,21,22,31,36–39].

What remains under-represented in the literature are RCTs with large sample sizes, RCTs for different training venues (e.g. in the home versus in hospital), follow-ups of more than three months, active versus standard-care control groups, evidence of a cause-and-effect relationship between training and participation outcomes and RCTs for specific populations (e.g. community-dwelling veterans with SCI, whose demographic and clinical characteristics as well as health-care experiences may be different from general populations [40–42]).

Our primary objective was to test the hypothesis that community-dwelling veterans with SCI who receive the WSTP in their own environments significantly improve their manual wheelchair-skills capacity in comparison with an Educational Control (EC) group. Our secondary objectives were to describe differences in the success rates for individual skills, to test the hypothesis that any improvements would be retained at one year, and to test the hypothesis that such training has an impact on participation.

## Materials and Methods

### Participants

We studied community-dwelling veterans with SCI who used manual wheelchairs, a sample of convenience. A power analysis was conducted using Cohen’s methods, conventions as implemented in Power Analysis and Sample Size (PASS) software [43] and assumptions based on the data of Routhier et al [30]. Sample size was chosen to provide at least 80% power to detect Cohen’s effect size for the primary objective (total and subtotal WST scores) midway between middle and small (incremental R^2^ = 0.07), tested with two-tailed two-sample t tests of pre- vs post-training change scores and a conservative Bonferroni-adjusted α of 0.0125. Calculations showed that 94 participants would be needed for such an analysis. We oversampled, in anticipation of drop-outs between the baseline and post-training assessments.

### Study Design

**Fig 1:**
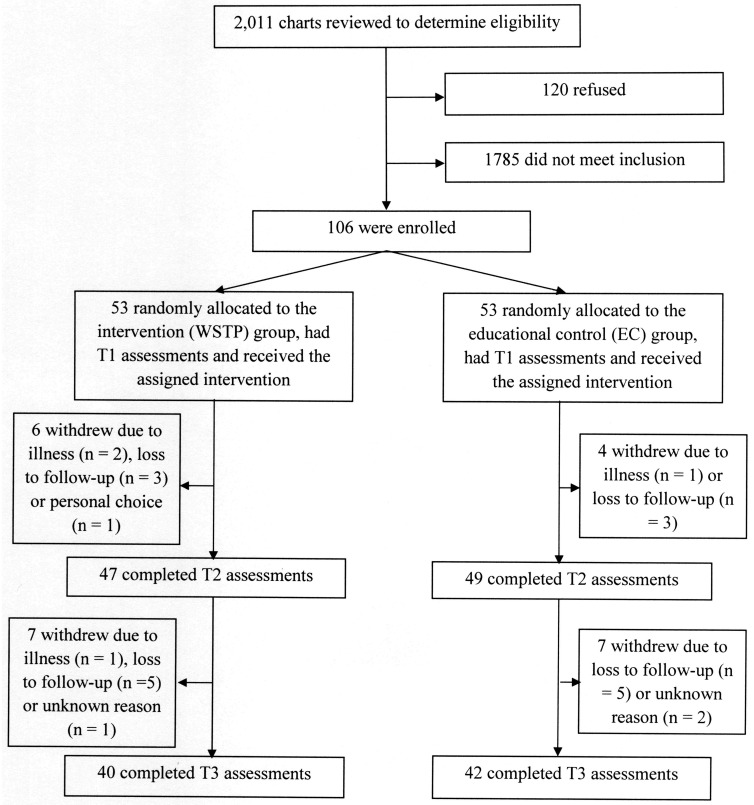
CONSORT flow diagram. This diagram illustrates the numbers of charts reviewed, the number of participants enrolled, allocated to each group and assessed at baseline (T1), after training (T2) and at one-year follow-up (T3).

An un-blinded RCT design was used for this multi-site study. CONSORT guidelines were followed (Fig 1) (see S1 File).

### Clinical Trial Registration

The Clinical Trial Registration Number is NCT00434018. The original protocol can be viewed in S2 File. There are restrictions prohibiting the authors from making the minimal dataset publicly available. Veterans Affairs is in the process of determining policy and procedures for publically sharing data. Until that time, Veterans Affairs investigators are prohibited from publically releasing data. The name of the individual who readers may contact to request the data is Dr. Gail M. Powell-Cope (Gail.Powell-Cope@va.gov). Data will be available upon request to all interested researchers who meet the VA policy guidelines.

### Sites

The three sites were the James A. Haley Veterans’ Hospital in Tampa, Florida, the Charlie Norwood Veterans Administration (VA) Medical Center in Augusta, Georgia and the West Roxbury campus of the VA Boston Healthcare System in Boston, Massachusetts.

### Ethical Issues

Ethical approvals for this study were obtained from the Institutional Review Boards (IRBs) of each of the three participating centers—the Institutional Review Board of the James A. Haley Veterans’ Hospital in Tampa, FL (IRB #006372), the Office of Human Research Protection, Human Assurance Committee (HAC), Medical College of Georgia, Augusta GA (HAC #0705290), and the Veterans Affairs Boston Health Care System IRB—Human Studies Subcommittee (IRB # 2030). Each participant provided his/her written informed consent prior to any study participation.

### Recruitment and Screening

Participants were recruited by recruitment flyers, word of mouth and review of health records for individuals who met eligibility criteria. Potential participants who met initial criteria were mailed letters informing them of the study and asking any interested individuals to contact the study coordinator for additional information. To ensure the privacy of potential participants, no identifying information was utilized until the participants provided consent to participate in the study.

### Inclusion and Exclusion Criteria

Each participant was a veteran, had a SCI for at least one year, had a level of injury at C6 and below, used a manual wheelchair as the primary means of mobility, was able to self-propel the wheelchair, was between the ages of 18 and 75 years, was living within 241 km (150 miles) of the research site, was able to follow simple instructions and was willing to participate (as manifested by providing informed consent and completing the baseline [T1] assessment). Potential participants were excluded if they had a progressive disease, had a cardiac or respiratory condition that limited physical performance, had any unstable medical conditions or were pregnant.

### Demographic and Clinical Data

To describe the sample, we collected demographic and clinical data at intake by chart review and interview. We recorded age, sex, SCI injury level, duration of SCI, number of comorbidities (e.g. hypertension, stroke), highest level of education completed, employment status, marital status, race/ethnicity and research site.

### Wheelchair Data

Participants used the wheelchairs that they ordinarily used. No alterations were made by study personnel to optimize fit or function. Wheelchair specifications were recorded at T1.

### Group Allocation

Participants were randomly assigned to either the WSTP or EC groups by using a computer-generated blocked randomization schedule. This was done to ensure that at no time during randomization was the imbalance large and that at certain points the number of participants in each group would be equal. At the end of baseline data collection, each participant was handed a sealed envelope that had the study-group assignment and the schedule for skills training or education.

### Wheelchair Skills Training Program

The WSTP Version 4.1 included 32 individual wheelchair skills (listed later) divided into three skill levels (Indoor, Community and Advanced) [44]. The Wheelchair Skills Program (WSP) skill set is representative of skills identified as important by wheelchair users and healthcare professionals [45,46]. Participants each received five one-on-one training sessions. The training was carried out in the participant’s home unless the skill that he/she wanted to work on required the training to be done elsewhere (e.g. on a family member’s staircase).

The trainers (all of whom were therapists or therapy assistants) were trained in WSTP administration. Wheelchair skills trainers familiarized themselves with the WSP website and received in-person practical training from the WSP developers. For any research personnel who joined the study later, the outgoing person and research coordinator at that site trained the incoming person.

**Fig 2:**
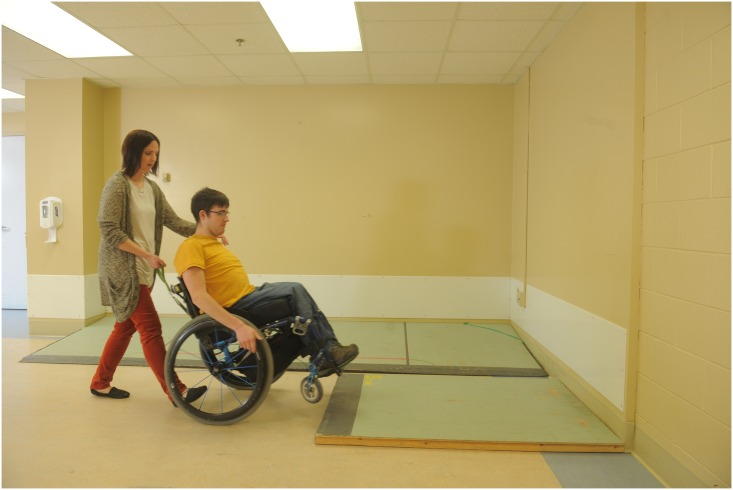
Example of wheelchair skill. The “ascends 5 cm level change” skill shown during the caster-popping phase.

**Fig 3:**
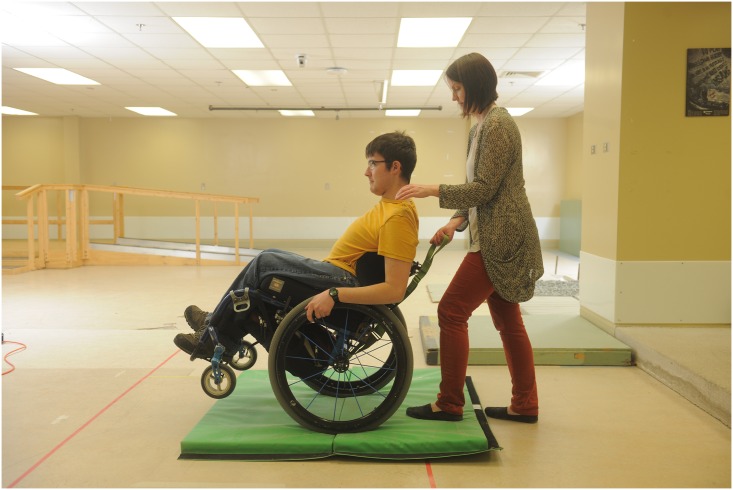
Example of wheelchair skill. The “stationary wheelie” skill being practiced on a soft surface before progressing to a smooth level surface.

**Fig 4:**
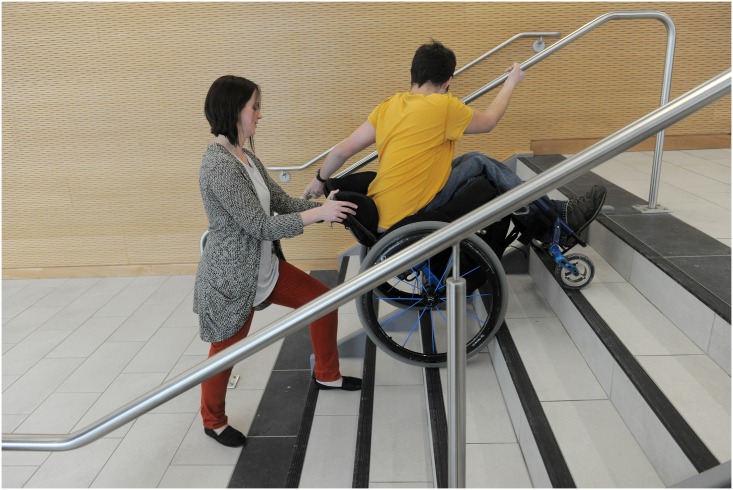
Example of wheelchair skill. The “descends stairs” skill using one of the options for hand positioning.

The initial participant training session provided the therapist with an opportunity to establish training goals based on the baseline evaluation of the participant’s skill level and his/her personal goals for training [47]. Examples of skills within the WSTP skill set are shown in Figs 2–4. The individuals shown in these figures have given written informed consent (as outlined in the PLOS consent form) to publish these photographs. Examples of training goals that fell outside the WSTP skill set were use of a customized vehicle lift system and transfer into a pool. Each training session was 30–45 minutes in duration. During training, whenever possible, a significant other or caregiver was present, to increase the likelihood of safe practice between the formal training sessions.

### Education Control

The EC intervention mirrored the WSTP in intensity, duration and process. The difference was in the content. Participants in the EC group received five home-based sessions about 45 minutes in duration that focused on health promotion for persons with SCI. The EC participants each had discussion with a research assistant (usually a nurse who worked on the SCI unit) on the topics related to general wellness after SCI, including nutrition, pressure ulcer prevention, prevention of infections, prevention of respiratory complications and the importance of exercise.

Education Control trainers received training from the research coordinators at each site. The material covered in the sessions was part of standard care for people with SCI and the research personnel were already well-versed in the content.

Using principles of adult learning, each session began with an informal pre-test. The trainer then used a printed “Fact Sheet”, 3–10 pages of information that was discussed with the participant. The same Fact Sheets were used at all sites and with all participants. Examples of the content of such sessions were the importance of maintaining strength and range of motion for health and function and the importance of frequent weight relief from the buttocks as a means of preventing pressure ulcers. The sessions were individualized based on the pre-test and the specific health issues of the participant. During training, whenever possible, a significant other or caregiver was present. The session ended with an informal post-test and the participant received printed materials to keep.

### Outcome Measures

#### Wheelchair Skills Test

The WST has been highly ranked in independent surveys of such tests [48,49] and it has been well studied with respect to measurement properties. The WSP website includes a dynamic link [50] that performs a customized and instantaneous search of PubMed literature; as of November 17, 2016, the link listed 54 published papers either specifically about the WST and its questionnaire version (WST-Q) or that had used these measures in studies. The WSTs for this study were carried out either in participants’ homes or the study hospitals. Each of the 32 individual skills was scored as a ‘pass’ (score of 1) or ‘fail’ (score of 0) for capacity [51] on the basis of defined evaluation criteria [52]. The total WST capacity score was the percentage of skills that were passed. Subtotal scores for the Indoor, Community and Advanced level skills were also computed. We followed the procedures of the WST 4.1 Manual [52]. All data collectors were trained in WST administration.

#### Craig Handicap Assessment and Reporting Technique (CHART)

A number of participation measures are available for people who use wheelchairs [5,6,53–55], from which we selected the CHART [54,55]. The CHART is a general measure of participation that captures the interaction of the person and the environment, community reintegration and participation. The CHART quantifies handicap by evaluating six domains: cognitive independence, economic self-sufficiency, mobility, occupation, physical independence and social integration. Each of the six subscales has a maximum score of 100, and the subscale scores were summed to form a total score (maximum of 600). High scores indicate lesser restriction in participation.

#### Participants’ Perceptions

We recorded any of the participants’ spontaneous comments that were of relevance to the training intervention.

### Procedure

The enrollment process consisted of having interested individuals contact the site project manager who verified eligibility criteria, answered any study-related questions and obtained contact information in order for research staff members to schedule an initial visit. During the initial visit, the research staff verified eligibility criteria, obtained informed consent and collected demographic, clinical and wheelchair data. The participants were randomized to the WSTP or EC group. Participants were provided with either wheelchair-skills training or education in their own environments over a 5-week period. Data were collected at three time points: baseline (T1), early post-intervention (T2, 4–5 weeks after T1) and 12 months post-intervention (T3). Scheduled phone calls every two months between T2 and T3 were used as a strategy to increase subject retention.

### Data Analysis

Teleforms (TeleForm v 10. HP Software Headquarters, HP Autonomy, 1140 Enterprise Way, Building G, Sunnyvale CA 94089–1412) were used for data entry, then data were verified for accuracy. Demographic, clinical and wheelchair data were reported descriptively for the T1 time point, comparing the two groups to assess comparability using Chi square for categorical data and two-sample t tests for continuous data. Two-sample t tests were used to compare the T1-T2 and T2-T3 latencies (in days) of the two groups. To assess whether there was a training effect due to the WSTP intervention, we used two-tailed, two-sample t tests to compare the groups’ change scores (T2-T1), initially using only data from the participants who completed the study. For these analyses, we looked at total and subtotal (Indoor, Community and Advanced levels) WST scores and total and subscale CHART scores. Additionally, we used repeated measurement Analysis of Variance (ANOVA) to assess the interaction between group and time and multivariate models that included the baseline demographic and clinical variables.

We assessed the effect of drop-outs by comparing the demographic and clinical characteristics of drop-outs with those who completed the study (using Fisher test for categorical variables and t tests for continuous variables) and by conducting Intention to Treat (ITT) analyses on the WST outcome variables. For the two ITT analyses, we replaced missing values with either the previous value or the mean value for that group at that time point.

To assess whether there was retention, we used paired t tests to evaluate the WSTP group with respect to the total and subtotal WST change scores (T3-T2). For each of the individual skills, we calculated the n (%) of participants in each group who were successful at each time point. We used a criterion of ≥20% difference between time points for our definition of a clinically significant difference (one of sufficient magnitude to induce a therapist to alter his/her standard practice) [25, 30]. We used <75% of the group being able to complete a task as a criterion of low success rate for that skill as Hosseini et al [24] and Kirby et al [25] have done.

All data analyses were completed in Statistical Analysis Software version 9.4 (World Headquarters, SAS Institute Inc., 100 SAS Campus Drive, Cary, NC 27513–2414, USA). A Type 1 error rate (α level) of <0.05 was used to define statistical significance. We elected not to Bonferroni-adjust the α level because the primary objectives were independent a priori; rather we reported the actual p values [56].

## Results

### Demographic and Clinical Data

The CONSORT [57] flow diagram is shown in Fig 1. In Table 1 are shown the demographic and clinical data at T1 for the 106 participants who enrolled in the study. The two groups were comparable with respect to the parameters shown. The average age of participants was in the late 40s, there was a very high predominance of males and over two-thirds of participants in both groups had SCIs at the thoracic level. The mean duration of the SCIs in both groups was over 15 years. The median number of comorbidities was low. About three-quarters of participants had completed at least four years of college education and about one-third were employed. About half were married or partnered and over three-quarters were white. About one-half of participants were from the Tampa site and the others were about equally divided between the Boston and Augusta sites. In comparing the characteristics of the 24 participants who dropped out with the 82 who completed the study, the only statistically significant difference was that a smaller proportion of the drop-outs (54% vs 84%) had more than four years of college education (p = 0.002).

**Table 1:** Demographic and Clinical Data at Baseline.

Parameter	Statistic	WSTP^a^ Group (n = 53)	EC^a^ Group (n = 53)	P-value
Age^b^	mean (SD)^a^	48.1 (13.6)	47.1 (12.6)	0.701
Sex^c^	Male	51 (96.2)	50 (94.3)	0.647
Level of SCI^a^^,^^c^	Cervical	4 (7.5)	9 (17.0)	0.158
	Lumbar	3 (5.7)	6 (11.3)	
	Thoracic	46 (86.8)	38 (71.7)	
Duration of SCI^a^ (years)	mean (SD)	16.6 (12.9)	18.2 (13.0)	0.521
Number of comorbidities^b^	median (IQR)^a^	1.00 (0.00–2.00)	1.00 (0.00–2.00)	0.759
Education^c^	>4 years of college	41 (77.4)	41 (77.4)	1.000
Employment^c^	Yes	19/52 (36.5)	17/53 (32.1)	0.538
Marital status^c^	Married or partnered	30 (56.6)	25 (47.2)	0.523
Race/ethnicity^c^	White	45 (84.9)	45 (84.9)	0.753
	Black	5 (9.4)	5 (9.4)	
	Hispanic	3 (5.7)	2 (3.8)	
	Other	0 (0.0)	1 (1.9)	
Research site^c^	Augusta	13 (24.5)	12 (22.6)	0.965
	Boston	15 (28.3)	16 (30.2)	
	Tampa	25 (47.2)	25 (47.2)	

^a^Abbreviations: EC = Educational Control, IQR = interquartile range, SCI = spinal cord injury. SD = standard deviation, WSTP = Wheelchair Skills Training Program.

^b^Mean (SD) scores are shown for continuous data where data were normally distributed, otherwise median (IQR) values are shown.

^c^For categorical data, n (%) values are shown.

### Wheelchair Data

The wheelchair specifications at T1 are shown in Table 2. There were no significant differences between the WSTP and EC groups. About two-thirds of the wheelchairs were rigid frame, about one-quarter were equipped with rear anti-tip devices, about two-thirds had armrests and almost half had air cushions.

**Table 2:** Wheelchair Specifications at Baseline.

Specification	WSTP^a^ Group^b^ (n = 53)	EC^a^ Group^b^ (n = 53)	P-value^c^
Rigid frame	38 (71.7)	30 (57.7)	0.133
Rear anti-tip devices	14 (26.4)	12 (22.6)	0.652
Wheel locks	47 (88.7)	48 (90.6)	0.750
Sling backrest	39 (75.0)	39 (73.6)	0.868
Rigid backrest	14 (26.9)	15 (28.3)	0.874
Foam cushion	13 (24.5)	9 (17.0)	0.338
Air cushion	25 (47.2)	24 (45.3)	0.846
Contour cushion	8 (15.1)	6 (11.5)	0.592
Other cushion	20 (37.7)	21 (40.4)	0.781
Adjustable armrests	11 (20.8)	10 (18.9)	0.807
Desk-length armrests	15 (28.3)	11 (20.8)	0.367
Full-length armrests	9 (17.0)	5 (9.4)	0.251
Removable front rigging	10 (18.9)	13 (24.5)	0.480
Swing-away front rigging	11 (20.8)	11 (20.8)	1.000
One-piece front rigging	40 (75.5)	43 (81.1)	0.480
Adjustable angle footplates	12 (22.6)	15 (28.3)	0.504
Positioning belt	2 (3.8)	1 (1.9)	0.558
Knapsack/backpack	26 (49.1)	19 (36.5)	0.195
Power add on	2 (3.8)	3 (5.7)	0.647

^a^Abbreviations: EC = Educational Control, WSTP = Wheelchair Skills Training Program.,

^b^n (%) values are shown.

^c^p-values are from two-sample t-tests comparing WSTP vs EC.

### Wheelchair Skills Test

The total and subtotal WST scores for the participants who completed the study are shown in Table 3. At T1, the mean total WST scores were high. The subtotal WST scores were high for the Indoor and Community levels and lower for the Advanced level. The median (interquartile range [IQR]) latencies for T1-T2 were 59 (49–93) days and 56 (42–67) days for the WSTP and EC groups (p = 0.0.035). The median (IQR) latencies for T2-T3 were 307 (290–320) days and 321 (301–357) days for the WSTP and EC groups (p = 0.052).

**Table 3:** Wheelchair Skills Test Data for Participants Who Completed the Study.

Parameter	WSTP^a^ Group^b^			EC^a^ Group^b^		
	T1^a^	T2^a^	T3^a^	T1^a^	T2^a^	T3^a^
n	53	47	40	53	49	42
Indoor	97.9 (6.1)	98.3 (5.2)	98.6 (4.9)	99.5 (2.1)	99.4 (2.2)	99.6 (2.0)
Community	94.1 (12.3)	95.3 (11.4)	96.1 (9.7)	95.4 (8.6)	95.3 (8.9)	97.9 (7.6)
Advanced	51.9 (26.9)	63.8 (29.8)	65.6 (33.2)	60.8 (26.7)	64.2 (26.5)	68.3 (29.8)
Total WST^a^	83.4 (12.7)	87.3 (12.8)	88.2 (12.8)	86.9 (10.3)	87.9 (10.0)	90.1 (10.8)

^a^Abbreviations: EC = Educational Control, SD = standard deviation, T1 = baseline, T2 = post-training, T3 = 1 year follow-up, WST = Wheelchair Skills Test, WSTP = Wheelchair Skills Training Program.

^b^Mean (SD) % scores are shown.

As shown in Table 4, the T2-T1 change scores for the total and Advanced-level WST scores were significantly higher for the WSTP group than the EC group based on the t tests. There were no significant differences in the T3-T2 change scores. These findings were also found for the ITT analyses, regardless of whether the last observation was carried forward or the missing values were replaced with mean values.

**Table 4:** Wheelchair Skills Test Change Scores.

Parameter	T2-T1^a^			T3-T2^a^		
	WSTP^a^	EC^a^	P-value^c^	WSTP^a^	EC^a^	P-value^c^
n	47	49	NA^a^	40	42	NA^a^
Indoor^b^	0.4 (5.7)	-0.2 (1.3)	0.493	0.7 (4.8)	0.0 (0.0)	0.358
Community^b^	1.4 (7.5)	-1.5 (7.7)	0.068	1.2 (11.2)	2.4 (6.3)	0.522
Advanced^b^	11.8 (14.0)	1.4 (12.4)	<0.001	1.4 (23.5)	2.4 (13.3)	0.814
Total WST^a^^,^^b^	4.0 (6.0)	-0.2 (5.0)	<0.001	1.1 (10.3)	1.6 (4.8)	0.777

^a^Abbreviations: EC = Educational Control, SD = standard deviation, T1 = baseline, T2 = post-training, T3 = 1 year follow-up, WST = Wheelchair Skills Test, WSTP = Wheelchair Skills Training Program.

^b^Mean (SD) values are shown.

^c^p-values are from two-sample t-tests comparing WSTP vs EC.

The same pattern of significant differences between the groups was seen when using the multivariate models for T2 vs T1 that included the baseline demographic and clinical characteristics except that the magnitudes of the differences were slightly higher—the mean (SD) total WST T2-T1 change scores were 5.9% (1.3) and 1.5% (1.2) for the WSTP and EC groups (p < 0.001); the Advanced-level subtotal WST T2-T1 change scores were 15.6% (3.1) and 4.4% (3.0) (p < 0.001). Although not statistically significant (p = 0.11), the Community-level change scores were also higher for the WSTP than the EC group, 3.0% (1.8) and 0.3% (1.7). There was no difference in this pattern for either of the ITT analyses.

Regarding retention of training benefits, in addition to the T3-T2 data shown in Table 4, paired t tests between the T3 and T2 data for the WSTP group revealed minimal increases (in the 0.3–1.9% range) at T3, none of which were statistically significant.

Individual skill success rates for the two groups are shown in Table 5. At T1 for both groups, there were 8 skills (7 of which were at the Advanced level) for which the success rates were <75% (our definition of a low success rate for a group). T2 success rates were ≥20% higher than T1 success rates (our definition of a clinically meaningful improvement) for two skills (*ascends 15 cm curb* and *performs 30 s stationary wheelie*) in the WSTP group only.

**Table 5:** Wheelchair Skills Test Individual Skill Success Rates.

Skill	Skill Level	WSTP^a^ Group^b^			EC^a^ Group^b^		
		T1^a^ (n = 53)	T2^a^ (n = 47)	T3^a^ (n = 40)	T1^a^ (n = 53)	T2^a^ (n = 49)	T3^a^ (n = 42)
1. Rolls forward 10m	Indoor	53 (100)	47 (100)	40 (100)	53 (100)	49 (100)	42 (100)
2. Rolls forward 10m in 30s	Community	53 (100)	47 (100)	40 (100)	53 (100)	48 (98)	42 (100)
3. Rolls backward 5m	Indoor	53 (100)	47 (100)	40 (100)	53 (100)	49 (100)	42 (100)
4. Turns 90° while moving forward	Indoor	53 (100)	47 (100)	40 (100)	53 (100)	49 (100)	42 (100)
5. Turns 90° while moving backward	Indoor	52 (98)	47 (100)	40 (100)	53 (100)	49 (100)	42 (100)
6. Turns 180° in place	Indoor	52 (98)	47 (100)	40 (100)	53 (100)	49 (100)	42 (100)
7. Maneuvers sideways	Indoor	53 (100)	46 (98)	40 (100)	53 (100)	49 (100)	42 (100)
8. Gets through hinged door in both directions	Indoor	50 (94)	46 (98)	40 (100)	53 (100)	49 (100)	42 (100)
9. Reaches 1.5m high object	Indoor	52 (98)	47 (100)	39 (98)	53 (100)	49 (100)	42 (100)
10. Picks object from floor	Indoor	52 (98)	45 (96)	40 (100)	53 (100)	49 (100)	42 (100)
11. Relieves weight from buttocks	Indoor	52 (98)	46 (98)	39 (98)	53 (100)	49 (100)	42 (100)
12. Transfers from wheelchair to bench and back	Indoor	49 (93)	43 (92)	36 (90)	50 (94)	46 (94)	40 (95)
13. Folds and unfolds wheelchair^c^	Community	10/15 (67)^e^	8/10 (80)	7/9 (78)	15/23 (65)^e^	16/22 (73)	15/16 (94)
14. Rolls 100m	Community	51 (96)	46 (98)	39 (98)	53 (100)	48 (98)	41 (98)
15. Avoids moving obstacles	Community	53 (100)	47 (100)	40 (100)	53 (100)	48 (98)	41 (98)
16. Ascends 5° incline	Community	52 (98)	45 (96)	40 (100)	53 (100)	49 (100)	42 (100)
17. Descends 5° incline	Community	52 (98)	45 (96)	40 (100)	53 (100)	48 (98)	42 (100)
18. Ascends 10° incline	Advanced	43 (81)	39 (83)	34 (85)	49 (93)	43 (88)	38 (91)
19. Descends 10° incline	Advanced	48 (91)	43 (92)	35 (88)	50 (94)	46 (94)	38 (91)
20. Rolls 2m across 5° side-slope	Community	52 (98)	46 (98)	40 (100)	51 (96)	49 (100)	41 (98)
21. Rolls 2m on soft surface	Community	52 (98)	46 (98)	39 (98)	53 (100)	49 (100)	40 (95)
22. Gets over 15cm pot-hole	Community	44 (83)	40 (85)	33 (83)	43 (81)	43 (88)	37 (88)
23. Gets over 2cm threshold	Community	53 (100)	47 (100)	39 (98)	53 (100)	47 (96)	41 (98)
24. Ascends 5cm level change	Community	43 (81)	42 (89)	36 (90)	47 (89)	44 (90)	40 (95)
25. Descends 5cm level change	Community	47 (89)	43 (92)	38 (95)	50 (94)	44 (90)	40 (95)
26. Ascends 15cm curb	Advanced	18 (34)^e^	26 (55)^d^	27 (68)^e^	27 (51)	28 (57)	25 (60)
27. Descends 15cm curb	Advanced	35 (66)^e^	36 (77)	28 (70)^e^	39 (74)	37 (76)	30 (71)
28. Performs 30s stationary wheelie	Advanced	29 (56)^e^	38 (81)^d^	29 (73)^e^	34 (64)	34 (69)	28 (67)
29. Turns 180° in place in wheelie position	Advanced	28 (53)^e^	30 (64)	25 (63)^e^	33 (62)	31 (63)	27 (64)
30. Gets from ground into wheelchair	Advanced	17 (32)^e^	20 (43)	17 (43)^e^	26 (49)	27 (55)	23 (55)
31. Ascends stairs	Advanced	11 (21)^e^	16 (34)	18 (45)^e^	13 (25)	17 (35)	20 (48)
32. Descends stairs	Advanced	18 (34)^e^	22 (47)	23 (58)^e^	19 (36)	20 (41)	22 (52)

^a^Abbreviations: EC = Educational Control, T1 = baseline, T2 = post-training, T3 = 1 year follow-up, WSTP = Wheelchair Skills Training Program.

^b^n (%) values are shown.

^c^The denominators are shown for the *folds and unfolds wheelchair* skill because there were missing values, due in part to the fact that some of the wheelchairs could not be folded.

^d^The success rate at T2 was ≥20% greater than that at T1.

^e^Success rate <75% at T1.

### CHART Scores

The total and subscale CHART scores for the participants who completed the study are shown in Table 6. At T1, the total and subscale CHART scores were high. There were no significant T2-T1 or T3-T2 differences in the CHART scores between the WSTP and EC groups based on the t tests or repeated-measures ANOVAs. Using the multivariate modeling for T2 vs. T1, only one of the six parameters was different to a statistically significant extent—the mean (SD) change scores for the Mobility subscale were 3.0 (1.8) and -0.7 (1.7) for the WSTP and EC groups (p = 0.021).

**Table 6:** CHART scores for Participants Who Completed the Study.

	WSTP^a^ Group^b^			EC^a^ Group^b^		
Scores	T1^a^ (n = 53)	T2^a^ (n = 47)	T3^a^ (n = 40)	T1^a^ (n = 53)	T2^a^ (n = 49)	T3^a^ (n = 42)
Cognitive Independence^c^	91.7 (13.9)	89.9 (14.8)	90.6 (13.1)	92.3 (13.7)	90.2 (14.3)	89.9 (16.8)
Economic Self Sufficiency^c^	86.9 (28.0)	79.9 (34.9)	75.3 (40.9)	84.0 (29.9)	85.2 (30.7)	87.2 (26.6)
Mobility^c^	93.0 (13.0)	94.1 (11.7)	93.3 (14.7)	93.4 (14.3)	91.6 (15.5)	90.7 (11.1)
Occupation^c^	69.2 (32.7)	74.6 (29.5)	70.6 (31.4)	72.1 (31.9)	74.4 (30.5)	71.8 (31.5)
Physical Independence^c^	91.4 (17.6)	89.9 (23.0)	89.5 (20.8)	93.0 (17.2)	93.0 (15.3)	92.1 (15.3)
Social Integration^c^	92.4 (15.8)	92.1 (16.1)	89.4 (16.6)	89.7 (16.9)	86.1 (25.8)	86.3 (20.9)
Total Score^d^	527.3 (64.0)	525.5 (73.6)	511.5 (85.6)	530.9 (68.8)	522.0 (71.7)	514.9 (59.1)

^a^Abbreviations: EC = Educational Control, SD = standard deviation, T1 = baseline, T2 = post-training, T3 = 1 year follow-up, WSTP = Wheelchair Skills Training Program.

^b^Mean (SD) values are shown.

^c^Sub-scores range from 0–100.

^d^Total scores range from 0–600.

### Participants’ Perceptions

The participants in both the WSTP and EC groups generally reported their experiences as being beneficial. The WSTP group participants’ comments included that they appreciated being able to personalize their goals, that they would have never attempted trying some of the skills if they had not had someone to work with one-on-one and that they were able to participate in the comfort of their own environments. A selection of representative quotes were: “I’ve conquered my fear”, “I feel so empowered”, “I am not afraid anymore to be alone” and “I don’t have to rely on others to help me”. Transfer from floor to wheelchair was indicated by our participants as one of the most important skills for them to learn. For the EC group, there was educational content that some of the participants said they had never heard before.

### Adverse Incidents

There were no adverse incidents affecting the participants during assessment or training activities. One trainer reported injuring his back while moving study equipment in and out of the van for a home visit. Reporting of the incident followed policy and the trainer was placed on light duty responsibilities during his recovery period.

## Discussion

The mean total WST scores were high, but roughly similar to those reported for non-veteran manual wheelchair users with SCI [21–25]. Of the three mean subtotal WST scores, the Indoor level was highest and the Advanced level the lowest, as has previously been reported by Routhier et al [30] and Worobey et al [32].

The hypothesis that training would increase WST capacity scores was corroborated for the total and Advanced-level WST scores. For the total WST scores, the mean absolute magnitudes of T2-T1 improvement (from the multivariate models) were 5.9% and 1.5% for the WSTP and EC groups respectively, corresponding to relative improvements over baseline of 7.1% and 1.7%. For the Advanced-level WST scores, the mean absolute magnitudes of T2-T1 improvement were 15.6% and 4.4% for the WSTP and EC groups respectively, corresponding to relative improvements over baseline of 30.1% and 6.9%. These relative improvements are similar in magnitude to previous WSTP training RCTs for other populations of manual wheelchair users [27–32].

There were slight improvements in the WST scores of the EC group. This phenomenon has been previously reported [27–32], but the explanation for this is unclear. One possible explanation is that participants in control groups, having attempted all of the skills in the WST, may have experienced some intrinsic learning (i.e. the WST itself may produce a training effect) [26]. Using the questionnaire version of the WST (the WST-Q) might have obviated this methodological problem.

In choosing to study community-dwelling veterans [40], we anticipated that there might be some demographic and clinical differences between them and previously reported cohorts of people with SCI [21–25]. Of these other cohorts, the two from the United States (US) SCI Model Systems group (Hosseini et al [24] on 214 participants and Kirby et al [25] on 117 participants) provide the most relevant comparisons. In comparison with the data of those two studies, our veteran participants were older, more were male, more were white, more were married or partnered, more had completed at least 4 years of college, more had SCIs at the paraplegic than tetraplegic levels and the durations of SCI were longer.

Supporting the hypothesis that any training-induced improvements would be retained at follow-up one year later, the within-group paired t test between T2 and T3 for the WSTP group showed no significant decline.

The success rates for individual skills were consistent with the findings for the total and subtotal WST scores. For the WSTP group, there were 2 (6%) of the 32 skills (*ascends 15 cm curb* and *performs 30 s stationary wheelie*, both at the Advanced level) that met our criterion of ≥ 20% difference in success rate between T1 and T2. For both of these, the T2 success rates were higher. No skills met this criterion for the EC group. There were no skills in either group for which the success rate dropped by ≥ 20% between T2 and T3, supporting the retention hypothesis. At T1, the success rates for 8 (25%) of the 32 skills (all but one at the Advanced level) were <75% (our criterion for a low success rate) for both the WSTP and EC groups. This is comparable with earlier US studies of people with SCI [24,25].

Given the extended time since their initial SCIs (mean of > 15 years), it is of interest that wheelchair skills can be taught or relearned even years after injury and that the participants made positive comments about their training experiences. This may be because the early phases of rehabilitation can be busy and wheelchair skills training is only one of many issues that require attention. Wheelchair users may have more time and be more receptive to wheelchair skills training after they are settled in their communities but our study suggests that there remains room for improvement many years after injury. Performing training in the home environment was effective and appreciated by the participants.

The CHART data provided insights about the participation levels of our participants. The six subscale scores and the total score were all high. In comparison with the Hosseini et al [24] CHART data for people with SCI, the scores for our participants were higher for the Mobility sub-score and the total score. The only statistically significant T2-T1 difference between the WSTP and EC groups in our study was for the Mobility subscale based on the multivariate models, with absolute change scores of 3.0 and -0.7 for the WSTP and EC groups respectively, corresponding to relative changes at T2 in comparison with baseline T1 scores of 3.2% and -0.7%.

### Limitations

There were a number of study limitations, some of which have already been mentioned. Although an initial sample size of 106 is fairly large for a training study on participants with SCI who were followed for one year after training, the moderate number of drop-outs likely reduced the power of the study. However, we explored whether missing data due to drop-outs skewed our findings. We compared the demographic and clinical characteristics of the participants who completed the study and those who dropped out and found only level of education to differ, with the drop-outs having a lower level of education. As for the ITT analyses, neither of the two methods used provided results that were appreciably different from the results of the participants who completed the study.

We did not ask participants about any wheelchair skills training that they had received before the study, either as components of their initial rehabilitation or subsequently from the VA or another source. However, the VA provides the full continuum of care to veterans with SCI, including initial comprehensive rehabilitation. Wheelchair training is typically provided as an integral part of initial rehabilitation [58]. Some veterans also get initial rehabilitation in the private sector before transferring to the VA. Over 8% of individuals in the SCI Model Systems database have veteran status at time of injury. Close to half of those use VA healthcare services by the end of the first year post-injury and more receive VA care in subsequent years [59].

We also did not assess the appropriateness or fit of the wheelchairs that our participants used. However a recent study reported that people with SCI with the VA as the payer source consistently received high quality and appropriately customized wheelchairs and the VA was the only payer group for which all beneficiaries received wheelchairs that met standard of care [60].

There was a slight statistically significant difference in the T1-T2 latencies (longer for the WSTP group) and an almost significant difference (p = 0.052) for the T2-T3 latencies (longer for the EC group). However, the magnitudes of the differences were small and it is unlikely that they affected the conclusions that can be drawn from the study. Although the demographic and clinical data of our participants differed slightly from those reported in other US studies of participants in SCI Model Systems sites [24,25], such differences were expected [40] and the response to training was similar in direction and slightly greater in magnitude than the previous SCI training study that trained participants in groups [32].

The failure to blind testers to group allocation was a limitation; blinding of participants to the nature of their training would not have been possible. The failure to blind testers was due to budgetary constraints. The assessments were often carried out in the participants’ home environments by the same persons who conducted the training. The objective nature of the main outcome measure (the WST) was felt to mitigate this limitation. In the future, if similar constraints are an issue, we recommend achieving tester blinding by using the WST-Q by telephone.

Although we did not collect data on the cost of the WSTP intervention as we used it, it is likely that home training is more expensive to the healthcare system than hospital-based training due to the travel time and costs. Also, one-on-one training is likely to be more expensive than group training [32] because of the high trainer-to-participant ratio.

Given the high initial WST scores, there may have been a ceiling effect. In future studies, we recommend using the WSP Goal Attainment Score (GAS) [26] as a complementary outcome measure to the WST and/or the WST-Q. The WST-Q has the additional advantage of allowing documentation of wheelchair-skills performance and confidence.

Future studies will need to address these limitations. Additionally, research explorations of the low prevalence [18–20] and dose of training [19,61] may provide insights on what could be done to eliminate any barriers to training (such as lack of education or lack of confidence of clinicians). Other specific veteran populations (e.g. those using wheelchairs due to amputation [18] or stroke [62]) should be carried out. Additional topics for future study are comparison of outcomes for veterans to non-veterans and the impact of manual wheelchair selection, configuration and adjustments (i.e. optimal fit) on manual wheelchair skills.

In addition to manual wheelchair skills training, there is growing evidence of the need for and effectiveness of powered wheelchair skills training [63,64]. Most of the studies to date have taken place on community-dwelling wheelchair users but it is likely that more focus should also be placed on people with recent injuries [27,46]. Extensive wheelchair skills training is more likely to be feasible for the veterans population than for the general public because the length of stay and resource limitations are less likely to be limiting factors in the veterans population. However, the findings of this study are consistent with the improvements seen in the general population of wheelchair users with or without SCI [27–32].

In spite of the limitations of the current study and the need for future work, this is the first study to demonstrate the effectiveness and safety of one-on-one wheelchair skills training in the home environment for community-dwelling veterans with SCI.

## Conclusions

The implications of our findings in general are that such training should be made available to any person with SCI who uses a wheelchair, even if the person has been using a wheelchair for many years. As one means of accomplishing this objective, there should be more focus on wheelchair skills training in professional schools. The need for and effectiveness of such training has been previously reported [65–69]. SCI systems of care in general (and the US VA SCI systems of care in particular) should consider implementing a policy that ensures wheelchair skills assessments and training are performed to the full extent possible both as part of the initial rehabilitation process and later as people with SCIs are followed throughout their lives.

Individualized wheelchair skills training in the home environment improves the advanced wheelchair skills capacity of experienced community-dwelling veterans with SCI by 30% over baseline scores although it has only a small impact on participation levels. These findings suggest the need to consider a more formal approach to wheelchair skills assessment and training within the VA systems of care.

## Supporting Information

S1 File CONSORT checklist. (PDF)

S2 File Trial protocol. (PDF)

## Abbreviations

ANOVA: Analysis of Variance
CHART: Craig Handicap Assessment and Reporting Technique
EC: Educational Control
GAS: Goal Attainment Score
ITT: intention to treat
IQR: interquartile range
RCT: randomized controlled trial
SCI: spinal cord injury
SD: standard deviation
T1: evaluation at first time period (baseline)
T2: evaluation at second time period (post-training)
T3: evaluation at third time period (follow-up)
US: United States
VA: Department of Veterans Affairs
WHO: World Health Organization
WSP: Wheelchair Skills Program
WST: Wheelchair Skills Test
WST-Q: questionnaire version of the WST
WSTP: Wheelchair Skills Training Program
