# Supplementary material for: Manual Wheelchair Skills Training for Community-Dwelling Veterans with Spinal Cord Injury: A Randomized Controlled Trial
Source: PLoS One. 2016 Dec 21;11(12):e0168330. doi: 10.1371/journal.pone.0168330 (PMC5176312; doi:10.1371/journal.pone.0168330)
Supplement: S2 File — (PDF) [file pone.0168330.s002.pdf]

I. RATIONALE FOR STUDY

The wheelchair is among the most common and important of rehabilitation devices, particularly for persons with mobility impairments, including persons with a spinal cord injury (SCI).[1] Despite advances in wheelchair technology, users continue to face significant limitations to mobility and increased risk associated with falls and over-use injuries of the upper extremities. Mobility limitations arise as a result of environmental barriers (e.g., rough terrain, stairs, or steep inclines).[2] In order to deal with these barriers, manual wheelchair users need to develop a set of specific wheelchair skills (e.g., turning around in tight spaces or negotiating a curb). Without these critical skills, wheelchair users may avoid these obstacles altogether, significantly limiting mobility, community participation, and independence in daily life.

Wheelchair use poses significant risks, associated with morbidity and mortality. The most common safety hazards are wheelchair tips and falls, [3] upper extremity pain, [4 5 6 7 8 9] and over-use injuries of the upper extremities of the shoulder[10 11 12 13 14] and wrist. [15 16 17 18 19 20] The long term effects of these injuries, result in limitations of upper limb function, with a snowball effect and resultant functional limitations in mobility and independence, further detracting from quality of life and increasing the burden of chronic illness, injury, and disability. [21 22 23] In addition to significant pain and functional limitations, wheelchair falls account for 68.5% of fatal accidents among manual wheelchairs users of all ages and disabilities.[24]

We propose to test an evidence-based wheelchair skills training program to optimize wheelchair safety and performance in individuals with SCI. The proposed training program incorporates emerging evidence on wheelchair biomechanics and motor-skills learning, and addresses recommendations in a new clinical practice guideline. [25] Given the difficulty in translating wheelchair skills learned in a therapy clinic with "real world" problems in the home and community post-discharge,[26] we are proposing to conduct the wheelchair skills training in and around the participants' home. The immediate goal is to enhance ability, performance time, safety, community participation, and quality of life, while minimizing physical strain. The ultimate goal is to reduce morbidity/mortality associated with wheelchair use and promote successful aging with a disability.

II. SPECIFIC AIMS

A. Objectives: The purpose of this 3-year randomized controlled clinical trial is to evaluate use of a community-based wheelchair skills training program (WSTP). Objectives include:

1. Determine the immediate and sustained effects of a WSTP on ability, performance time, and physical strain.
2. Examine the effects of mediating and moderating variables on ability, performance time, and physical strain.
3. Examine the effects of a WSTP on wheelchair safety, community participation, and quality of life.
4. Promote dissemination of WSTP in VA SCI Centers through two key activities: (a) Design innovative marketing plans to increase participation and promote patient-centeredness based on patient-perceived benefits. (b) Estimate health care resources needed to add home-based wheelchair skills assessment/training in VA SCI Centers, nationally.

B. Hypotheses:

H1: Subjects who receive the WSTP will have significant improvement in immediate outcomes (ability, performance time, and physical strain) post training when compared with subjects in an Education Control (EC) group.

H2: The effects of the WSTP will be sustained over time (1 year).

H3: Subjects who receive WSTP will have significant improvement in community participation and quality of life when compared with subjects in the EC group.

H4: Subjects who participate in WSTP will have significant improvement in wheelchair safety, after adjusting for the extent of wheelchair use and risk-taking behavior.

C. Research Question:

Q1 What is the relationship between the intervention, and ability, performance time, and physical strain in persons with SCI, after controlling for demographic, mediating, and moderating variables?

III. BACKGROUND. The review of the literature is divided into four critical areas: (A) Risks Associated with Wheelchair Use, (B) Gaps in Existing Wheelchair Skills Training, (C) Evidence-Based Wheelchair Skills Training, and (D) Conceptual Framework.

A. Risks Associated with Wheelchair Use*:* Wheelchair use poses significant risks. More than 100,000 wheelchair-related injuries were treated in emergency departments in 2003; twice as many as were reported in 1991.[27 28 29 30] Wheelchair tips and falls accounted for 65-80% of injuries across all age groups of wheelchair users, and 17.2% of those injuries required admission to hospitals.[31] The most commonly cited *environmental risk factor* reported by subjects with SCI was the natural environment. [32] More than twice as many accidents occurred outdoors than indoors, and curbs, ramps, stairs, uneven terrain, and wet or icy surfaces have been implicated.[33 34 35 36 37 38] *Risky wheelchair activities* include transfers (41%), reaching for an object (33%), propelling over uneven terrain (11%), movement in bed (17%), operating a van lift (4%), and using a shower chair (4%). Factors contributing to these fall-related activities included loss of balance, equipment failure, spasticity, pain, and excessive speed.[39]

Poor wheelchair skills contribute to wheelchair tips and falls, the most common form of incidents.[40 41 42] Tips and falls account for 68.5% of fatal and 73.2% of nonfatal accidents among non-institutionalized users of manual wheelchairs of all ages and disabilities.[43] In a study of 577 community-dwelling wheelchair users, 57.4% reported falls at least once during the time they had used wheelchairs, which averaged 11 years, and 66.0% reported having experienced a wheelchair tip. Of the falls and tips reported, 46.3% were in the forward direction, 29.5% were backward and 24.2% were sideways.[44] The body parts affected by the injuries in this group include head and neck (50%), trunk (9.5%), wrist and hand (9.1%), shoulder (8.1%), hip or thigh (8.1%), knee/leg (6.7%), ankle/foot (5.2%). Studies that reported serious injuries associated with wheelchair falls, lower extremity fractures accounted for 93-97% of the injuries.[45] A serious fall-related injury includes concussions, serious head injuries, fractures, dislocations, amputations, or spinal injuries.[46] Three different studies have estimated the yearly incidence of *serious* wheelchair-related accidents as 1%, 3%, and 17.7%.[47 48, 49]

Poor wheelchair skills contribute to upper extremity pain, injury, and dysfunction. Persons with SCI must learn to perform activities of daily living, such as mobility, weight transfer, and repositioning without the functional use of their lower extremities. Many years of overuse of the upper extremities lead to an increased incidence of cumulative trauma to the upper extremities. *Upper extremity pain* includes discomfort to the shoulder, elbow, wrists, and hands, with the shoulder as the most common injury site reported in SCI. Cross-sectional studies reported SCI shoulder pain prevalence at 51%, [50] 31%,[51] and 36%.[52] One study using self-reports linked shoulder pain with paralysis and duration of injury, exclusive of age.[53] In two studies using radiography of the shoulder, degenerative changes were seen in 32% [54] and 71% [55] of persons with SCI. Carpel tunnel syndrome is a common neurologic cause of upper limb pain in persons with SCI; prevalence was documented from 49-73%. [56 57 58 59]

B. Gaps in Existing Wheelchair Skills Training: Most rehabilitation facilities teach wheelchair skills to some extent, but significant variation exists in the content and process of the training. Few facilities have significantly changed their approaches to wheelchair skills training over the past decade, despite (1) changes in wheelchair technology, (2) new evidence on wheelchair biomechanics, and (3) new clinical practice guidelines.[60] In addition to the content and process, the timing of wheelchair skills training has long been acknowledged to be a problem.[61 62] Training typically is included during initial rehabilitation but a gap exists between what is taught in acute rehabilitation and the patients' actual performance at home.[63] Reasons for this gap include (1) fatigue, stress, and recovery from trauma interfere with a persons ability to learn in acute rehabilitation, (2) shorter lengths of stay limit time available to cover the wide range of skills needed for living with a disability (e.g., bladder and bowel care, self-care training);[64] (3) difficulty translating wheelchair skills learned in a therapy clinic with "real world" problems upon discharge,[65] and (4) delays in ordering the wheelchair and having it available for the patient to have enough supervised practice time. In summary, new wheelchair users are likely to reenter the community with suboptimal wheelchair skills.

Post-rehabilitation, many factors interfere with wheelchair skills training over the course of one's life. Persons with SCI obtain new wheelchairs over time, but may not receive additional training, even though wheelchair technology continues to evolve. New wheelchair technology includes design features that offer patients complicated tradeoffs between safety and efficiency. For example, wheelchairs that provide variable rear axle position offer the advantage of efficiency in propelling by placing the axle close to the system center of gravity; however, the risk for tipping over backwards increases.[66, 67] The use of smaller, harder front wheels maximizes maneuverability, but also contributes to forward tips and falls by increasing susceptibility to tips when propelling over uneven terrain or stopping abruptly.[68, 69] The use of camber (angling the rear wheels so that the bottoms of the wheels are farther apart than the tops) makes wheelchair propulsion easier, but is associated with a three fold increased risk of tipping over, because it moves the center of gravity of the occupied wheelchair backward and causes a backwards tilt of the frame.[70,71] Wheelchairs have been designed to be lighter, making wheelchair propulsion easier, but this also increases the risk of tipping, especially when a knapsack is placed on the back of the chair to use to carrying objects while propelling.[72, 73] Further, as people age, wheelchair skills that were previously effective, may cause problems; new strategies are needed to compensate for comorbidities, weakness, or frailty. The long term effects of poor wheelchair skills are significant. Inability to maneuver, upper extremity pain or injury, limits community participation and detracts from quality of life.

C. Evidence-Based Wheelchair Skills Training*.* Our research team is currently studying wheelchair falls in SCI [IIR –03-003-1]. Through observations and interviews, we have identified wheelchair skills training as the most promising *modifiable* intervention to promote safety and performance. Clinicians at the Tampa, Augusta, and Boston SCI Centers have identified a "practice gap" in both the quality and consistency in which wheelchair skills training is provided. To bolster wheelchair skills training received in acute rehabilitation, continued training after community reentry may be a sensible strategy. After acute rehabilitation when individuals experience a wide range of barriers in the natural and built environment, they may better recognize the need for such training. Although there are excellent wheelchair training resources in textbooks and training manuals, there are very few reports of the effects of wheelchair-skills training in the research literature, [74 75 76 77 78 79] and none specific to younger, more active SCI patient populations. In controlled settings, wheelchair skills training increased performance and participation in wheelchair users. [80 81 82 83 84] However, these studies need to be replicated in "real life" settings. Existing research on wheelchair skills training, although supportive of the hypotheses of this proposal, used heterogeneous samples that may not generalize to SCI.

Newly published clinical practice guidelines[85] suggest that wheelchair propulsion techniques and resulting forces on the upper extremity contribute to wrist and shoulder injury. The guidelines recommend teaching wheelchair users optimal propulsion techniques to reduce potentially injurious wheelchair push forces; specifically, wheelchair users should use long smooth strokes when propelling their wheelchairs on the level, recovering their hands below the hand rims.[86] Kirby et al.[87] describes the importance of the wheelie in manual wheelchair use, an event that occurs when the front wheels, ordinarily in contact with the support surface, are intentionally caused, by means of a transient or sustainable rear pitch, to lift from the surface while the rear wheels remain on the surface. Although this type of maneuver is the foundation of many wheelchair activities, most wheelchair users never learn to perform this important skill.

Using methodology based on the extensive motor-learning literature, Kirby et al.[88] developed the WSTP.[89] The WSTP includes three skill levels (indoor, outdoor and advanced), and includes 57 individual wheelchair skills. In a randomized controlled trial (RCT) on *an inpatient rehabilitation unit*, a significant improvement was reported in wheelchair skills performance of individuals who completed WSTP compared to a standard rehabilitation program.[90] Likewise, in another RCT of *community based manual wheelchair users*, similar improvement was noted in the WSTP group compared to a control group.[91] This study included training in and around the participants' homes. In another RCT, on occupational therapy students, the WSTP group had significantly greater improvement in wheelchair skills and retained the skills 9-12 months later compared to a group that received a standard occupational therapy curriculum.[9293] While these studies support the practicality, safety and efficacy of the program, the sample sizes were small and the sample was heterogeneous in both age and impairment. None focused on SCI.

In an inpatient SCI patient population, the longitudinal relationship between physical ability and wheelchair skill performance revealed a significant improvement of ability score (0.92 ± 0.13 $p<0.001$).[94] Another study looking at the relationship of manual wheelchair skill performance scores and community participation in SCI patients reported a significant association of ability, performance time and physical strain scores with community participation (adjusted R-squares varying from 0.20 to 0.26).[95] Building on these small studies, we will focus on community-based SCI population of veterans.

D. Conceptual Framework. The conceptual framework for this study is based on the World Health Organization model of functioning, disability/ health, and behavior change theory (Figure 1). The Institute of International Classification of Diseases and Functioning and Disability includes: (a) *Body functions and structure* instead of disease or condition; (b) *Activities* (related to tasks and actions by an individual) and *community participation* (involvement in a life situation);[96] and (c) the influence of *environmental factors* for determining disability.[97]

The person/environment fit is critical in understanding individual adaptation to disability.[98] The interaction of the environment and the person determines the degree of enablement or disablement,[99] defined as the degree of ability to perform socially defined activities and roles expected of individuals within a social and physical environment. This view of ability/disability is dynamic. It is

Figure 1. Conceptual Model for Testing the Effects of Wheelchair Skills Training on Patient Outcomes

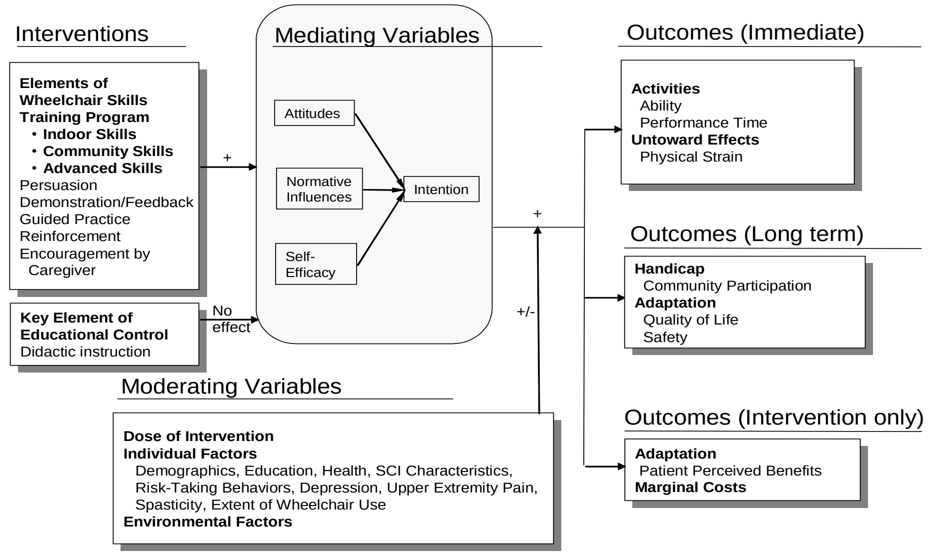

a product of the strengths and vulnerabilities of persons and of the supportiveness of the environment. It consists of three components: pathology (disruption of function or structure at the cellular level), impairment (loss of structure or function at the organ or organ system level) and function (restriction of lack of ability to perform an action or activity in the manner or within the range considered normal). This model depends on both personal change strategies and supportive environments to achieve health, though in the general health promotion field, many believe that supportive environments have the greater impact in producing lasting change.[100] Patrick[101] proposed that quality of life, as defined by the person with a disability, is the ultimate outcome of interest in disability outcomes research. He further proposed that quality of life is a function of the person/environment interaction and the proximal outcomes, or opportunity outcomes (e.g., freedom from injury) that allow full participation in society (community participation).[102]

Community participation is defined by the International Classification of Functioning, Disability and Health (ICF) as "involvement in life situations" (including work, housekeeping, social relationships, and community interactions), is an important rehabilitation outcome for persons with SCI.[103] Mastering wheelchair skills can make the difference between dependence and independence, supporting the evidence that wheelchair skill training is a critical element of the rehabilitation process. [104] [105] [106] Manual wheelchair skill performance in persons with SCI is positively correlated with community participation.[107] Wheelchair skills have been linked to community integration.[108]

According to a recent Institute of Medicine report, "The most critical determinant of whether a person does or does not perform a given behavior is the person's beliefs about performing that behavior,"[109] and thus an important variable that mediates the effects of any health promotion message or program. All of the most commonly applied behavioral change theories stress the importance of beliefs and <u>intention</u> to perform a behavior (or commitment to change) including Social Cognition Theory,[110] the Theory of Planned Behavior,[111] the Health Belief Model, and the Transtheoretical Model of Behavior Change.[112] Although different theories use different terminology, three major factors appear to influence intention (and thus behavior) including (1) one's <u>attitude</u> toward performing the behavior; (2) one's perception of the <u>norms</u> governing performance or nonperformance of the behavior; and (3) one's sense of personal agency or <u>self-efficacy</u> regarding personally performing the behavior."[113] All three of these constructs are represented in our model. The intervention was designed to foster positive attitudes about performing safe wheelchair skills, strengthen the normative influences of performance of wheelchair skills, and strengthen self-efficacy, or the individual's belief that he/she can perform wheelchair skills safely.

E. Work Accomplished to Date: Investigators at the Tampa Patient Safety Center of Inquiry (PSCI) have a comprehensive program of research related to safe patient mobility, including wheelchair safety. The following key accomplishments contributed to this study:

(1) In October 2000, the Tampa VA center received a $3.2 million grant to evaluate evidence-based fall interventions at 40 VAMCs. [114,115,116,117,118] Efforts focused on falls in ambulatory veterans living in the community. Despite the success of this program, questions were raised about the applicability of the program for wheelchair users. Given the large number of veterans who use a wheelchair for mobility, the lack of evidence-based practices was identified as a significant gap. Efforts to implement changes in wheelchair features were initiated, but this intervention alone was not effective in reducing fall-related injuries. Wheelchair safety was identified as the most important follow up study.

(2) During this same time period, fall experts from the USA and Canada participated in a research agenda-setting session sponsored by the Tampa PSCI, using a consensus-validation

process developed at the NIH. While several priorities were identified, the gap in science related to wheelchair safety and falls was identified.[119]

(3) In August 2001, we began to investigate falls in SCI wheelchair users. A chart review of records identified 45 persons with SCI treated for a fracture at the Tampa VA between1990-2000.[120] A telephone interview was conducted to determine if the fracture was associated with a fall and if so, to obtain information about the natural history of the fall, etiology, extent of injuries, and non-VA healthcare utilization. Over 83% of the fallers with fractures were admitted for inpatient stays, with a mean length of stay of 66 days per patient. The retrospective review provided some insights into the etiology of serious fall-related injuries, but did not address the more frequently occurring non-injurious falls, and near falls (tips). Wheelchair skills was the primary *modifiable risk factor* identified. In addition to our findings, our research team summarized the evidence on wheelchair-related falls.[121]

(4) Under the direction of Lee Kirby, MD (Co-Investigator) the team at Dalhousie University, in Halifax, Nova Scotia has been studying wheelchairs for over two decades. Using methods ranging from epidemiologic to laboratory-based designs, this team has made significant scientific contributions to wheelchair safety and performance, as evidenced by its 50 wheelchair-related publications (www.wheelchairskillsprogram.ca). This new understanding has led to changes in wheelchair training techniques, changes in international standards for wheelchair stability testing and new considerations in wheelchair design. With particular relevance to the current proposal, much of his work over the past 11 years has been on the development and evaluation of the assessment and training modules that make up the Wheelchair Skills Program. In four studies, three of which were randomized controlled, researchers concluded that this wheelchair skills training program was efficacious, safe, and practical. [122] [123] [124] [125]To date, the program has not been tested in SCI, nor in the VHA. Dr. Kirby has been a co-investigator with our research team for the past 3 years examining wheelchair falls in veterans with SCI.

(6) Our research team obtained funding from HSR&D for IIR –03-003-1, entitled Epidemiology and Cost of Falls in Veterans with a Spinal Cord Injury. This 3-year cohort study includes baseline data collection and monthly follow-up over a 12-month period. The sample includes 720 SCI veterans, recruited from Tampa, Augusta, and Boston VAMCs. Data collection from this study is nearly complete. We are creating models for predicting wheelchair tips, falls, and fall-related injuries in this population. We found that 25% of subjects reported wheelchair falls and 12% reported fall-related injuries. In our sample of 704 subjects from 3 VA SCI Centers, we found a surprisingly large number with sub-optimal wheelchair skills. This study also included a qualitative component; data revealed that SCI veterans could be categorized as “Risk Takers” or “Risk Managers”. Risk takers were those who lead an active or athletic lifestyle, and who do not make changes in their activities after a fall. Their meaning of life was derived from their ability to engage in high risk activities that they are not willing to give that up even though they may have to endure pain and some loss of function due to repeated injuries over many years. They tended to be relatively young. They viewed safety wheelchair features as “unnecessary” and limiting. They viewed falls as an evitable part of life. On the other hand, risk managers, led more sedentary lives, were more likely to either change their lifestyle after a fall, and were more likely to use protective wheelchair equipment. Although they, too, viewed wheelchair falls as inevitable, they were more likely to use active prevention strategies.

Based on these preliminary results, we plan to use this data for social marketing of the proposed wheelchair skills program. We will design flyers and brochures that appeal to both groups. For example, we will not focus on wheelchair fall prevention, which has little appeal to either group. Rather, for the risk manager type, we would focus on wheelchair safety and preventing long term consequences of poor wheelchair skills. On the other hand, this would not

appeal to the larger, younger group of “Risk Takers”; we will recruit this group through brochures and flyers announcing a program for “Extreme Wheelchair Skills Training”. Despite variations in marketing techniques, the program goal, content and process will be the same for both groups. These evidence-based social marketing approaches will enhance recruitment.

F. SIGNIFICANCE /RELEVANCE. This study will be one of the first to address modifiable risk factors contributing to morbidity and mortality associated with long-term wheelchair use. An estimated 2.3 million Americans rely on wheelchairs to compensate for mobility impairments.[126] Wheelchairs typically are used 365 days a year for an average of 16 hours per day.[127] Falls and overuse injuries of the upper extremities are high-frequency, high-risk problems. The most common safety hazards are wheelchair tips and falls, [128] upper extremity pain, [129 130] and overuse injuries of the shoulder[131 132] and wrist. [133 134] Wheelchair tips and falls account for 68.5% of fatal accidents among manual wheelchairs users.[135]

SCI care is a priority for the VA. VA is the largest single network of SCI care in the nation. VA's SCI System of Care's mission is to support, promote, and maintain the health, independence, quality of life, and productivity of individuals with SCI throughout their lives. [136] Because the average age at time of injury is 32 years, specialized care is life-long. Persons with SCI have increasingly longer life expectancies and, as they age, they risk developing secondary conditions. The estimated national economic impact of SCI is about $9.73 billion per year. There are many gaps in knowledge of SCI treatment and management, and research efforts are needed to address these gaps in an effort to lead to improved outcomes, and gain a better understanding of factors that impact health, function, and quality of life. VHA has made a commitment to provide the specialized quality patient care by furnishing the most appropriate equipment and wheelchairs to disabled veterans and has stressed a special commitment in this regard for veterans with special needs such as SCI at considerable cost. Yet optimal use of wheelchairs is hampered by inadequate training.

Healthcare costs associated with pain and injuries due to wheelchair use are significant. From FY 98-01, 125,859 injury discharges occurred within the VHA system with a primary diagnosis for an injury incurring $1,547,308,636 dollars in costs.[137] The Decision Support System (DSS) cost data demonstrated that for every injury discharge prevented, there is significant cost avoidance estimated at $16,142 per patient in the VHA (in FY 02). These savings reportedly would accrue, on average, irrespective of the mechanisms of injury, are likely to be higher for persons with SCI.[138]

Our key stakeholders for dissemination, the National Center for Patient Safety, the Chief Consultant for Spinal Cord Injury and Disorders Strategic Healthcare Group, Paralyzed Veterans of America, National Consortium for Spinal Cord Injury Medicine Clinical Practice Guidelines, and the SCI QUERI, have voiced strong opinions that practice changes are more likely to occur if the problem is costly and there are clear benefits for the recommended practice changes. Cost-effectiveness makes a convincing argument to both VA and non-VA administrators for adopting policy and practice changes. Cost data from this study will be used to document the direct costs associated with wheelchair skills training and build a business case for implementing the program within the 23 VA SCI Centers nationally. Dr. Nelson serves on the SCI QUERI Executive Committee as well as the Steering Committee for the National Consortium for Spinal Cord Injury Medicine Clinical Practice Guidelines. Both groups are very interested in translating findings from this study into practice across all 23 VHA SCI centers.

G. Value Added/Expected Products: Comprehensive preventive health evaluations are offered annually to veterans with SCI, using a specific approach outlined by VA policy.[139] The scope of the evaluation is comprehensive and includes multiple preventive health elements and SCI specific evaluations. While required annual evaluation elements include rehabilitation functional assessments and education about prevention of musculoskeletal problems, they currently omit testing and reinforcing of wheelchair skills. Our experience, corroborated by evidence from our recent study, suggests that wheelchair skills testing and/or training is typically not included in the SCI annual evaluation. [We recruited over 700 SCI veterans immediately after their annual evaluation; no deficits in wheelchair skills were documented in the medical record after the annual evaluation, however these same veterans performed poorly in wheelchair skills testing just hours later, suggesting a performance gap.] The annual evaluations offered for persons with SCI have by no means been a static program and have been evolving. It is expected that as standards of care are developed or studied, they will be implemented in the SCI System of Care and in the SCI annual evaluations. Recent reports[140] suggest that certain tests and laboratory evaluations currently performed as part of the SCI annual evaluation may be of limited utility, and that it may be prudent to replace those elements of the annual evaluation with other evidence-based practices that have a demonstrable effect on the health, function, and independence of veterans with SCI. At the end of this study, we will be able to provide the following products:

1. Evidence-based recommendations for incorporation of wheelchair skills testing and training as part of the life-long SCI continuum of care offered by the VA. Data from this study will provide a basis for both VA and non-VA administrators and clinicians to make policy and practice changes. Our preliminary discussions with the VA SCI Strategic HealthCare Group indicate that they are strongly supportive of this initiative and intend to use data from this study for practice change throughout the VA SCI System of Care.
2. We will adopt an evidence-based wheelchair skills training protocol that has been adapted for use in the VA, which includes the content, process, and expected outcomes.
3. This study will report effects of wheelchair skills training in a population of wheelchair users with SCI. The few studies conducted to date have mostly included a mixed population of wheelchair users, across age and impairment groups; these studies have relied on small sample sizes. The proposed study will have sufficient power to answer the questions posed. By focusing on SCI, we may be able to tease out unique aspects of the program applicable to this younger, more active, risk-taking population.
4. While we are focusing on persons with SCI in this study, the findings may be relevant to other impairments and disabilities as well. Findings will be exported throughout the VHA through the Chief Consultant for Spinal Cord Injury and Disorders Strategic Healthcare Group, National Consortium for SCI Clinical Practice Guidelines, and the SCI QUERI.

H. DESCRIPTION OF THE INTERVENTION. The WSTP is based on the motor-learning literature,[141] [142] [143] [144] [145] and was designed by Dr. Kirby (Co-I) and his team.[146] The WSTP includes 3 skill levels (indoor, outdoor and advanced) comprising 57 individual wheelchair skills. Subjects will receive 3 evaluation study visits and 5 sessions primarily in their home environment, with a few sessions that may take place during routine hospital visits or at the therapists' discretion. The curriculum is based on the participant's goals and baseline abilities (as documented using the Wheelchair Skills Test [WST]). The trainer will be a therapist or therapy assistant who has been trained and certified in WSTP procedures. The sessions will be 30-60 minutes in duration, to avoid fatigue and overuse symptoms. The training will take place in and around the participant's home, although stairs training may be completed in the therapy clinic. Experience with training in the community to date suggests that obstacles appropriate for the testing and training can usually be found within a short

radius of the participant's home. Each session will begin with a brief warm-up and finish with a brief cool-down and the assignment of homework to be practiced before the next session. During the main body of the session, the participant will begin by practicing skills that he/she can already perform, but has only recently learned (e.g., in the previous session). These skills will be practiced in random order and in a variety of settings. Such variation is known to enhance skill retention and transfer. Then the trainer will work with the participant on skills that he/she is still attempting to acquire. In addition to intrinsic feedback (the participant's own recognition of an attempt's success or failure and why that might be), feedback from the trainer will be provided, beginning with a 50% feedback schedule (i.e., after every 2nd attempt) and weaning gradually. Because Knowledge of Results (KR) is usually obvious (e.g., was the attempt to ascend a curb successful or not), the feedback content will more often be Knowledge of Performance (KP) (e.g., the reason for failing to get up the curb was that the casters were popped off the ground prematurely), particularly prescriptive KP (identifying the single action that the participant should do differently to improve safety or performance). It is important not to overload the participant with too much feedback. While acquiring a new skill, segmentation of serial skills will be used. Skill difficulty will progress gradually (e.g., low → medium → high curbs). After working on a complex new skill, to enhance consolidation, no additional complex skills will be introduced for at least 6 hours, preferably not before the participant has slept. This is why such new skills are only practiced late in each training session. Once a skill has been acquired, it will be added to the repertoire of skills that the participant will practice in subsequent sessions, in random (rather than blocked) order and in a variety of settings (to enhance skill retention and transfer). The Wheelchair Skills Program Manual can be downloaded from: www.wheelchairskillsprogram.ca. During training, whenever possible, a significant other/caregiver (e.g., family member or friend) will be present. The caregiver will be trained to serve as a spotter, so that the participant will be able to safely practice the skills that he/she is working on between sessions with the trainer. The WSTP trainer and the designated caregiver /significant other will serve as spotters for skills for which there is a risk of the participant tipping over or falling from the wheelchair. For skills during which there is a risk of the wheelchair tipping over backwards or running away, a spotter strap will be used. Spotting procedures are included in the WSP Manual and are part of the Train-the-Trainer program.

The Education Control Group will mirror most aspects of the WSTP, in intensity, duration, process, and trainers. The difference will be in the content. Subjects in the EC group will also receive 5 home-based follow up sessions; their sessions will focus on health promotion for persons with SCI. Session #1 will focus on nutrition, targeting the unique needs of persons with SCI. Session #2 will focus on respiratory health. Session #3 will focus on exercise, targeting the unique needs of persons with SCI. Session #4 will focus on hand hygiene and preventing infections. Session #5 will focus on preventing pressure ulcers. (See Appendix for content). The trainer for the EC will be a clinician with SCI experience, including nursing staff (RN, LPN, or NA) or therapists (including therapy aides) who has been trained and certified in EC procedures (typically 10 -hour training program, followed by supervised practice and an examination). Sessions will be 30-60 minutes in duration. The trainer will use a printed topical "Fact Sheet" of information that will be discussed with the subject. The sessions will be individualized based on the pre-test given at baseline, and specific health issues of the subject. For example on nutrition, weight loss may be the focus of discussion for some subjects, while others may need discussion on nutritional supplements or increasing dietary fiber. The EC training will take place in the subject's home. During training, whenever possible, a significant other/caregiver will be present, to reinforce the training. The subject will receive printed materials to keep.

IV. METHODS

A. Study Design. A two group randomized placebo controlled design will be used for this study. Participants with SCI will be assigned randomly (randomization process described later) to either the intervention WSTP or an EC group. Historically, ethical issues have limited the use of the randomized controlled clinical trials for at risk populations when withholding an intervention known to be of benefit to study subjects. Our answer to this ethical dilemma exists in the study design. Both WSTP and the EC will be provided in the home and will take the same amount of time thereby controlling for the effect of time spent attending to subjects across groups. Should the intervention be effective, subjects in EC will be able to enroll in wheelchair skills training after completion of the study (wait listed for these interventions). While we considered blinding data collectors to group assignments, budgetary limitations impeded our ability to hire additional staff necessary to complete this. Data will be collected at three points in time: baseline, immediately post-intervention (4-5 weeks), and 12 months post intervention. These three data collection points will allow us to assess the immediate and long-term effects of the intervention. Data collection strategies will be triangulated and include direct observation/performance testing, medical record review, and questionnaires. Figure 2 depicts the study design.

Figure 2: Study Design

	Baseline	Intervention	1 month	1 year
**Randomization**	O1	X	O2	O3
	O1		O2	O3

Addressing Threats to Validity. The observed effect of an intervention (e.g. WSTP) can be caused by several factors other than the direct effect of the intervention including: (1) natural course of the disease or process; (2) Hawthorne effect, the observation that the attention of being studied influences responses, (3) placebo effect, the phenomenon that a patient's symptoms (wheelchair skills) can be improved by an otherwise ineffective intervention, since the individual *expects* or *believes* that it will work, and (4) training to the test. The proposed randomized placebo control study design will allow us to control each of these threats to the study validity. First, the randomized control group design controls for the effects of the natural course of the disease because improvement from this will occur in both groups equally. Secondly, the control group (EC) controls for the Hawthorne effect because both groups will receive the same amount of attention. However, it is worth noting that this effect would only lead to an underestimation of the differences between the intervention and control groups, making it more difficult to corroborate the hypotheses. Thirdly, to eliminate the placebo effect, the design utilizes a placebo (education) control. Also, the placebo effect is less likely to influence an objective measure like the Wheelchair Skills Test than a subjective measure. Lastly, training to the test will be minimized by conducting the training in and around the home, and adding a long term evaluation, which occurs 1 year post training.

B. Sample: *(1) Inclusion Criteria:* (1) SCI for at least 1 year (neurologically stable)[*], (2) level of injury: C6 and below who use manual wheelchair as a primary means of mobility, (3) able to self-propel wheelchair, (4) between the ages of 18-75, and (5) able to follow simple instructions. *(2) Exclusion Criteria*: (1) progressive disease (e.g. spinal tumor), (2) extended bedrest for more than 30 days, (3) ventilator-dependent, (4) any cardiac or respiratory condition that would limit

[*] While WSTP would be useful for newly injured persons with SCI, we are excluding them since neurologic instability may interfere with evaluation outcomes. We also believe the WSTP would need to be modified to meet their unique needs, and offering different training modules to subjects based on function would contaminate the evaluation. Further, a portion of this training would be completed in the hospital, not in the home.

subject's physical performance, (5) unstable medical conditions, (6) use power wheelchair or scooter as primary means of mobility, † and (7) pregnancy. Although this research is being conducted at VA medical centers, subjects do not have to be veterans in order to participate.

C. Sample Size

(1) Sample Size Quantitative: We reviewed the literature for similar studies to prepare a power analysis. To facilitate comparison across different studies, we used the results in terms of standardized effect size (m1-m2/pooled standard deviation) as described by Cohen.[147] A RCT[148] reported a significant improvement (11.2 $\pm$ 10.2 $p < 0.000$) in wheelchair skills performance of wheelchair users admitted in rehabilitation patients who underwent WSTP, as compared to the standard rehabilitation program. In a RCT of community based manual wheelchair users, significant improvement of WSTP score (11.8 $\pm$ 12.95) in WSTP group was reported when compared to the control group. [149] Another randomized controlled trial, on occupational therapy students, found that the WSTP resulted in significantly greater improvement (9.8 $\pm$ 8.51) in wheelchair skills than a standard undergraduate occupational therapy curriculum and that these skills were retained 9-12 months later. [150] A study by Kilkens et. al. [151] looking at the longitudinal relation between physical capacity and wheelchair skill performance in SCI patients undergoing active rehabilitation, reported a significant improvement of ability score (0.92 + 0.13 $p<0.001$). Another study looking at the relationship of manual wheelchair skill performance scores and 'participation' in SCI patients reported a significant association of ability, performance time and physical strain scores with participation (adjusted R-squares varying from 0.20 to 0.26). [152] Based on the results described earlier we anticipate a substantial improvement (large effect size) in wheelchair use, community participation and quality of life for participants in WSTP as compared to the EC at time 2 (one month post-intervention) in the proposed study. We expect this difference to persist over time (12-months), but there is a possibility that this difference between the two groups might deteriorate over time. To ensure that an adequate sample size will be obtained if the effect size is smaller, a conservative strategy will be employed. We first considered using a Cohen's small effect size but were concerned that such differences would not be clinically relevant. Alternatively we considered the medium effect size but were concerned that this was not conservative enough. We settled on an effect size mid-way between the small and medium effect size as being a reasonable compromise. Table 1 shows estimates of sample sizes for small, medium and mid-point effect sizes. Sample size requirements were calculated for the regression analysis.

Table 1: Sample Size

	Small Effect Size	Mid-point Effect Size	Medium Effect Size
Partial R2	0.02	0.07	0.13
Sample Size per group	254	68	38
Total Sample Size	508	136	76

Power analyses were conducted using Cohen's methods and conventions as implemented in Power Analysis and Sample Size (PASS) software.[153] Sample size was chosen to provide at least 80% power to detect Cohen's effect size midway between middle and small (incremental R^2=0.07), tested in linear model analyses with alpha of 0.05, two-tailed using a maximum of 15 variables. Calculations using PASS show that 136 subjects are needed for such an analysis. This

† While WSTP would be useful for persons who use powered chairs, we are excluding them because (1) use of manual and power wheelchairs is very different and the skill sets for each are dramatically different—we prefer to evaluate one program first and follow up with a powered WSTP, (2) manual wheelchairs are more commonly used, and (3) the assessment and training protocols vary by type of chair. For example, the Smart Wheel is designed for manual wheelchairs only. Measurement tools for ability and strain would also vary considerably for powered wheelchairs. A future study is planned for powered wheelchairs.

adjustment is based on our experience with our study on wheelchair tips and falls in SCI patients at 3 VAMCs, where we had a 22% attrition rate over a 12 month period.

Repeated measures design is efficient in determining an intervention effect and consequently a smaller sample size is generally required.[154] Repeated measurements are usually positively correlated as they pertain to the same individual. A study with moderate correlation between baseline and follow-up measures and high correlation within follow-up measures with one baseline and two follow-up measurements would require 80% of the number of patients for a study with just one baseline and follow-up.[155] Therefore our repeated design will give us more power to detect our targeted effect size and will even allow us to detect an even smaller effect size if it exists. Because adding a mixed effect in linear regression model is expected to give a better fit, we expect our sample size to be adequate for the mixed effect analysis.

D. Subject Recruitment Subjects will be recruited through recruitment posters in VA elevators and in clinic waiting rooms (See Appendix), study brochure, and/or potential subjects will be referred by their primary care physicians. We will also ask Paralyzed Veterans Association (PVA) to post invitations in their newsletters and offices. (See discussion under work accomplished to date for overview on recruitment approaches, designed from our previous work). In addition, a mailing to SCI outpatients will be conducted. Potential subjects will also be approached by the data collectors and given the flyer and study brochure to review. Members of the study team with access to CPRS may also search for subjects who are eligible for this study by checking for inclusion/exclusion criteria in this VA patient database. To ensure the privacy of the patients, no identifying information will be asked or collected from the patient until they consent to participate in the study. Conversations pertaining to participation in this study will only be with the participants themselves. In addition, efforts will be made to recruit non-VA participants including individuals from consumer groups like sport organizations and from local SCI clinics in hospitals. Individuals from the study team will make flyers and brochures available to interested individuals and may attend functions to be available for recruitment and questions from potential participants. To proactively address potential problems with subject recruitment, the investigators and research team will meet on a regular basis (every two weeks initially, then monthly) to monitor recruitment goals for the study and take corrective action early if goals are not met. A *Recruitment Contingency Plan* will be developed, in the event of problems to ensure that recruitment lags do not occur.[156]

Recruitment Contingency Plan – Developed as of November 2009

As indicated in the section above, members of the study team will make efforts to recruit non-VA participants. To address this, local SCI outpatient clinics like Tampa General Hospital (TGH) and Bay South will be asked to make our flyers/brochures available to their patients. Members of the research team will attend local SCI support group meetings and local clinic staff meetings to present information about our study and be available for questions. Additionally, magazines and merchant catalogs intended for individuals with disabilities will be asked to advertise study information (approved publication information). For example, Disabled Dealer Magazine which is distributed to both dealers of wheelchairs and wheelchairs users has offered to post our study information in their upcoming editions. Companies who provide disability products/services will be asked to include our study brochure in their next mailing or to do a mailing of our study information to their customers. If a company agrees to do a mailing, they will be asked to include a letter on their company letterhead indicating that they support this research project, but participation in research project is based solely on the individual and will not affect their service from the company should they choose not to participate. The companies will also

include our study brochure/flyer and our research letter asking that interested individuals contact a member of the study team.

For all the recruitment methods listed in this Contingency Plan, no member of the research team will initiate direct contact with potential participants. Supplies for mass mailings will be provided to the companies so that the study information will come directly from them and not our research center.

Contingency Plan – Developed as of March 2010
To assist with recruitment of new subjects, we will offer all new subjects enrolled as of April 1, 2010, financial compensation of $15 for each evaluation visit completed. No payments will be provided for training visits or uncompleted evaluation visits. There are three (3) evaluation visits for each subject, totaling $45 worth of compensation to those who are eligible.

E. Subject Enrollment: The enrollment procedure includes: (1) Subjects interested in participation will contact the Project Manager. The project manager or designee on the study staff will respond to the patient within 24-48 business hours to determine if the subject is eligible for participation. (2) If eligible, the subject will be evaluated either in his/her home or at the VA (Visit 1) where the study will be described and the subject will be asked to sign an informed consent. As part of the consent process, the subjects will be asked if they would give permission to be photographed and/or videotaped at some point during their participation in the study. These images will possibly be used in presentations and publications. Subjects will be told that they can decline to be photographed and/or videotaped and that their choice will not affect their participation in the study. (3) During Visit 1, baseline data will be collected, and a pre-test will be administered to subjects which will include items addressing nutrition, preventing infection, pressure ulcers, respiratory health, and preventing infection (4) The subject will be randomized to a study group. (See randomization protocol described below). (5) The subject will be given a schedule of training (WSTP group will receive wheelchair skills training; EC will receive education training on SCI health promotion). Both groups will receive their respective trainings primarily in the home setting.

F. Subject Retention: Retaining subjects in a large-scale study can be problematic, particularly with multiple training visits and three data collection points. We added features to enhance subject retention. The investigators and research team will meet on a regular basis (every two weeks initially, then monthly to enable updates concerning retention of subjects for the study. This will ensure good communication between all concerned, and in the case that subject retention is a problem, solutions can be developed prior to major problems. This technique has been shown to be effective in retaining subjects for research studies.[157] Should retention problems be identified, a *Retention Contingency Plan* will be developed. [158] Further, we will initiate the following strategies: (1) A study brochure will be provided to subjects with specific details regarding the study, such as keeping appointments, what to do if they are unavailable or are out of town for an extended period of time, and who to contact, should there be questions; (2) A certificate of training completion will be provided to the subjects at the end of the course, and a thank you note after each data collection point to let patients know their efforts are appreciated. These procedures have been shown to be effective in retaining subjects for research studies.[159] (3) Reminder phone calls will be provided to subjects one or two days before home visits to reduce no shows; (4) Brief bimonthly phone contacts are planned as a recruitment strategy found to be effective in longitudinal studies; and (5) We will minimize subject burden by traveling to the subject's home for the intervention and qualitative interviews, making every effort to minimize length of data collection sessions.

G. Variables/Measurement: Table 2 outlines each variable, including definition, source of data, subject burden, and frequency of data collection. Immediately following the table, each tool is briefly described; the tools are included in the Appendix.

Table 2: Measurement Plan

VARIABLES	DEFINITION	SOURCE OF DATA	BURDEN	FREQUENCY
**Dependent Variables**				
Ability	Total Ability Score: The numerator is the Total Raw Score (i.e., the number of individual skills awarded a passing score) and the denominator is the number of applicable skills (i.e., the total number of skills [57] minus those awarded NP scores). 100% is the maximum possible percentage score. [Indoor, Community, Advanced]	WST	27 minutes	Baseline, Post Training, and 1 year
Performance time	Ability to move quickly in specific situations – a timed propulsion trial to simulate crossing a street.		< 5 minutes	
Physical Strain	Upper extremity loading measured by peak forces applied to the left and right pushrim of the subject's wheelchair during three standardized maneuvers.	Smart Wheel	15 minutes	
Community Participation	How often a person gets around their community; it includes all sub-components of the CHART	CHART	5 minutes	
Quality of life	Self perceived health status as measured by the SF-36V	SF 36V	15 minutes	
Wheelchair Safety	WST Safety Score: the percentage of attempted skills that were performed safely, regardless of whether the skill was passed or failed	WST	0	
**Mediating Variables**				
Attitudes	Overall evaluation of the performance of wheelchair skills	Attitude and Beliefs Questionnaire	5 minutes	Baseline, Post Training, and 1 year
Normative Influences	Perceptions that people who are important to the person think he/she should or should not perform the behavior in question.			
Self-Efficacy	Confidence in ability to master wheelchair skills			
Intention	Likelihood that an individual will engage in a given behavior, e.g. wheelchair skills as instructed			
**Moderating Variables**				
Dose of intervention	Hours of participation in actual training plus hours of self-report practice.	Activity Logs	< 1minute	Each session
Demographics	Age, Gender, Ethnicity	Demographic Data Form or Medical Record	0	Baseline Only
Education	Highest level of education completed.			
Health	Comorbidities, BMI			
SCI Characteristics	Level of Injury, Completeness of injury, Duration of injury (years)			
Risk Taking Behavior	Defined as sensation seeking, the tendency to pursue novel and stimulating experiences. Those high in Sensation Seeking have strong positive affective reactions to situations of novelty and risk, are sensitive to internal sensations and choose environments that augment them.	Zuckerman Sensation Seeking Scale	15-20 minutes	
Extent of Wheelchair Use	Distance (meters) traveled per week and average velocity.	Wheelchair Data Logger	5 minutes to attach datalogger	Baseline, Post Training, and 1 year
Depression	Amount of depressive symptoms experienced in previous month.	CES-D	5 minute	
Upper extremity pain	The severity of present and past shoulder, elbow, wrist and hand pain and its functional effect on wheelchair use and transfers.	VAS	< 5 minutes	

VARIABLES	DEFINITION	SOURCE OF DATA	BURDEN	FREQUENCY
Environmental Factors	A function of the frequency with which an environmental barrier is present and the degree to which it presents a problem for the individual. Includes the following barriers: attitude and support, services and assistance, physical and structural, policy, work, school.	CHIEF	10 minutes	
Spasticity	Severity of spasticity past and present; not limited to upper extremities as spasticity anywhere can affect wheelchair use, e.g., transfers.	VAS	< 5 minutes	

Wheelchair Skills Test (WST) version 4.1[‡] will be used to measure ability, performance time, and safety. The WST is an instrument for the objective evaluation of wheelchair skills. The WST consists of a series of commonly used wheelchair skills spanning the spectrum from those as basic as applying brakes to those as difficult as climbing curbs and performing wheelies. The WSC encompasses 57 skills (in Version 4.1) which result in a total score. The WST provide a pass-fail score for each skill. Refusal to attempt a skill (e.g. because of fear) constitutes a failing grade. The numerator is the Total Raw Score (i.e., the number of individual skills awarded a passing score) and the denominator is the number of applicable skills (i.e., the total number of skills minus those awarded NP[§] scores). 100% is the maximum possible percentage score. The WST has been demonstrated to be safe, and well tolerated with very good to excellent test-retest (0.65 p =.001), intra-rater (0.96 p =<.001), and inter-rater reliability (0.95 p = <.001) and content and construct validity. [160,161,162] Additional evidence of the construct validity of WST has been provided by studies that have used the WST as an outcome measure[163,164,165,166,167]. The mean time to perform the WST has been found to be less than 30 minutes.

- Performance time is part of the WST requiring subjects to propel their wheelchair for a given distance which simulates crossing a street in a timely fashion. Subjects are timed. It is felt that individuals who have better wheelchair skills will be faster in performance time.
- Wheelchair Safety is calculated as the percentage of skills that were attempted and performed safely, regardless of whether the skill was passed or failed.

The *Smart Wheel* will be used to measure physical strain. The SmartWheel is a wheelchair based measurement system that allows for the collection of propulsion kinetics. [168] It is a commercially available force-and torque-sensing push-rim that has been used in several studies, including those conducted in subjects with SCI,[169,170] to examine three-dimensional (3-D) propulsion forces (peak resultant force, wheel torque, mechanical effective force, and maximum resultant force rate of rise), moments, and temporal characteristics. Strain will be assessed by determining the maximum force exerted on the wheel while propelling compared to the maximum forces exerted on the pushrim while wheels are locked. The SmartWheel is integrated into a 24" wheel and is easily mounted to the vast majority of manual wheelchairs much like a quick-release wheel is taken on and off. Adapters are available for the attachment of SmartWheels to other manual wheelchairs that may not be in the 'vast majority.' It will be mounted to the right side of each subject's wheelchair. The subjects will be asked to go from a resting position (hands in lap and wheelchair stopped) to pushing at a self-selected comfortable speed over three different surfaces: 1) level tile; 2) up a 5-degree tile ramp; and 3) over medium pile carpet. SmartWheel data will be collected during the entire time. Researchers at the Pittsburgh VA have collected this data on over 100 subjects with SCI; all variables can be calculated without a kinematic system. These propulsion variables have been found to be reliable, repeatable,[171] and related to injury.[172]

‡. This is the latest version at the time of the proposal. Version 4.0 is in development and will be used if available when the study commences.
§ "NP: No Part – Wheelchair does not have the component"

A *Wheelchair Datalogger* will be used to measure extent of wheelchair use**.** The datalogger is capable of measuring distance traveled, speed, and time of movement. [173 174] The datalogger is weather proof, tamper resistant, and can be easily mounted onto the individuals' wheelchair. A small pendulum mounted inside the enclosure behind the PC board accomplishes motion sensing. A 0.125" diameter magnet pressed into one end of this pendulum; maintains a 6 o'clock position. Whenever the wheel and enclosure rotate, the magnet sweeps in a circle over six sensors mounted at sixty degree intervals behind the PC board; the sensors trigger an ultra low power microcontroller (Texas Instruments, MSP430F149). This microcontroller maintains a real-time clock and tags each sensor event with a time stamp. The time stamp records the time of each pulse to the nearest tenth of a second. This allows us to determine distance, speed, and time and date when activity occurred. The data are compressed and stored on the serial flash memory chip. Flash memory will retain data even if there is a power failure. A rugged Delrin cap fits tightly over the front of the aluminum enclosure and is sealed by a neoprene O-ring. Two tiny LEDs on the front of the PC board flash to provide status information; a 0.250" diameter Lucite window in the Delrin cap permits these LEDs to be viewed without disassembly. A lithium battery powers the WMDL; which mounts above the PC board. Data reduction is completed in MATLAB, and is easily inputted into statistical analysis programs. We will collect data for two weeks following each assessment. At the time of the assessment, a datalogger will be attached to the individual's wheelchair. After two weeks, subjects will be instructed to remove the datalogger and send it back to the investigators in the shipping materials provided. At that time, the data from the datalogger will be downloaded to MATLAB, and then processed to calculate average weekly distance and average velocity for the two-week period.

The *Craig Hospital Inventory of Environmental Factors—Short Form (CHIEF-SF)*[175 176 177] will be used to measure environmental factors. The CHIEF-SF is designed to assess the frequency and magnitude of perceived physical, attitudinal, and policy barriers that keep people with disabilities from doing what they want or need to do. It is designed to be an inventory of environmental barriers that can be utilized in large-scale surveys and surveillance systems, and be valid for both individuals with and without disabilities. The CHIEF-SF consists of 12 items. The individual is asked to evaluate the frequency with which barriers were encountered on a scale from 0 (never) to 4 (daily), then for each barrier, determine the magnitude of the problem on a scale from 0 (no problem) to 2 (big problem). A frequency-magnitude product is calculated for each item indicating the overall impact of the barrier. Extensive testing of the full instrument showed high levels of test-retest reliability, marginal subject/proxy agreement and evidence for between group validity. Factor analysis for the 25-item scale yielded five factors: attitude and support barriers, services and assistance barriers, physical and structural barriers, policy barriers, and work and school barriers. Access to the environment and has been shown to be positively and linearly associated with satisfaction with life. [178] Analysis of the CHIEF-SF supported these five subscales.

The *Craig Handicap Assessment and Reporting Technique (CHART)* will be used as a measure of handicap that captures the interaction of the person and the environment, and of community reintegration and participation. The CHART quantifies handicap by evaluating five domains: physical independence, mobility, occupation, social integration, and economic self-sufficiency.[179],[180] The CHART is made up of 27 questions with responses that are countable or in a yes/no format. Each of the five subscales has a maximum score of 100, and they may be summed to form a total score. High scores indicate lesser handicap. The CHART was developed as a specific instrument to measure handicap, is the only measure of that concept validated specifically for persons with SCI, and is currently the most widely used measure related to community reintegration in SCI. Reliability and validity of the CHART have been examined in multiple studies, and have been consistently found to be high. Norms are available for different lesion levels.

Short Form-36V (SF-36V) will be used to assess health status. The SF-36V181 is a modification of the SF-36, a generic health status measure that has been shown to be valid and reliable in a wide variety of health care settings.[182,183] The SF-36V modifies the SF-36 for use in VA ambulatory care populations by increasing the number of response options for items measuring role items thereby reducing problems with floor and ceiling effect in the two sub-scales measuring role functioning. The SF-36V consists of 8 scales including physical functioning, role-physical, bodily pain, general health, vitality, social functioning, role-emotional, and mental health, yielding two summary scores, physical health and mental health.[184,185] Teleform

Attitudes and Beliefs Questionnaire will be used to measure attitudes, normative influences, self-efficacy, and intention**.** Items from each of these scales will be summed to yield summary scores.

- *Attitudes* will be measured using 10 five-point items anchored by bipolar adjectives that patients commonly use when discussing wheelchair skills training, e.g. beneficial/harmful, useful/useless, convenient/inconvenient, physically stressful/stress free.
- *Self Efficacy* will be measured using two items: one five-point scale anchored by the bipolar adjectives of under my control/outside of my control and one item tapping into confidence in performing skills. Items will be summed to yield a total score.
- *Normative Influences* will be measured by a 3-item scale. Participants are asked to rate how likely three referents (primary care provider, friends/family, most people who are important to me) are to support them in performing wheelchair skills. Each item is weighted by how the individual us influenced by each person/group. The measurement approach for attitudes and beliefs and normative influences is based on a wealth of theoretical support,[186] [187] [188] empiric support[189] [190] [191] [192] and as well as past work by members of the research team. Using this methodology, we have developed similar scales to measure attitudes in health research, and achieved consistently reliabilities of greater than .80 for most scales, and evidence for content and construct validity.[193] [194] [195] According to experts on the Theory of Planned Behavior, the ideal process for developing these instruments is to conduct qualitative interviews with individuals representative of the population of interest, conduct content analysis, identify the most salient beliefs and attitudes, translate the beliefs and attitudes into survey items, then test the items. Because this process is resource intensive and time consuming, we drew on our knowledge of the subject matter and experience in skills training and survey item development to construct a draft questionnaire for the proposed study. This questionnaire is in the process of being pilot tested with 20 persons with SCI and reviewed by 10 external experts in SCI to evaluate content validity, and inter rater reliability. We will make revisions based on the pilot testing.

Therapist Activity Logs and Subject Activity Logs. The therapist/therapy aide will record the dose of intervention, operationalized as the hours of participation in training (WSTP or EC) for each subject. In addition, subjects will be required to keep a practice log of the time they spend on practice each day.

The *Zuckerman Sensation Seeking Scale* will be used to measure risk-taking because high sensation-seekers tend to be high risk-takers. Sensation Seeking is defined as the tendency to pursue novel and stimulating experiences. Those high in Sensation Seeking have strong positive affective reactions to situations of novelty & risk, are sensitive to internal sensations and choose environments that augment them. Sensation Seeking is a trait or state. The shortened version of the Zuckerman Sensation Seeking Scale is a 19-item questionnaire that yields four 10-item subscales (Thrill and Adventure, Experience Seeking, Disinhibition, and Boredom Susceptibility). The tool has established validity and reliability and is consistent across gender (except scale for Boredom Susceptibility).[196]

The *Center for Epidemiologic Studies Depression Scale (CES-D)* will be used to measure depression and taps into symptoms of depression in community populations. The scale consists of 20 items and are summed to yield a score. Scores range from 0-60; higher scores indicate more severe symptoms. Psychometric properties of this tool have been tested quite extensively and found to be good to excellent. Internal consistency as measured by Cronbach's alpha is high across a variety of populations (generally around 0.85 in community samples). Test-retest reliability studies ranging over 2-8 weeks show moderate correlation (r = 0.51 – 0.67) which is desirable for a test of symptoms that are expected to show change over time. Studies of African American versus Anglo-American versus Mexican American respondents show no differences in measures of internal consistency reliability.[197] Correlations with other depression scales have been found to be high in a variety of populations.[198]

Visual-analog scales (VAS) will be used to measure upper extremity pain and spasticity. Visual analog scales are a well accepted method for measuring pain and other individual symptoms. This method, which is useful for evaluating variations in pain intensity, the patient is instructed to indicate the intensity of his or her upper extremity pain by marking a 100-mm line with 2 extremes: no pain and worst imaginable pain. The VAS is based on the theory that pain intensity is continuous, without jumps or intervals. Many studies have supported the reliability and validity of the VAS as a sensitive measure of pain,[199] including several in patients with SCI.[200] It is generally accepted that the best judge of the severity of the spasticity and pain is the patient who is the person who can best assess its impact on his/her daily life.[201] The visual analog scale (VAS) is also used to measure self-rated quantification of spasticity in SCI[202 203] ranging from "no spasticity" to "most intense spasticity".

A *Demographics form* will be used to capture self-reported demographic data, comorbidities and SCI characteristics related information such as level and duration of injury. Should subject not know answers to these questions the Computerized Patient Record System (CPRS) will be accessed by the data collectors to collect this information.

A Health Related Survey (*Educational Pre- and Post-test)* will be used to measure subject's knowledge of health-related issues encompassing preventing infection, respiratory health, prevention and treatment of pressure ulcers, nutrition, and exercise. These tests will be in multiple-choice format and will be administered at baseline, 1 month follow-up, and 1 year follow-up.

H. Randomization procedure: The Project Manager will randomize the eligible subjects to one of the two groups (WSTP [Group 1] or EC [Group 2]) using a computer generated blocked randomization schedule. Blocked randomization will guarantee that at no time during randomization will the imbalance be large and that at certain points the number of subjects in each group will be equal. To preserve the unpredictability we will also randomly vary the block size to reduce the chances of the assignment schedule being seen by those responsible for recruitment of participants.[204] At the end of baseline data collection, patients will be handed a sealed envelope that will have their study group assignment and skills training/education schedule.

I. Data Collector Training: The data collectors will receive 40-60 hours of training before beginning data collection; this includes general hospital orientation and competency assessments. The therapist/therapy aide will be trained and certified in WSP procedures, in the standardized Train-the Trainers Program of the WSP (typically 10 -hour training session, followed by supervised practice and an examination). A training session will be held at the start of the study to train the therapists/therapy aides, the data collectors (inter and intra rater

reliability) on the study protocol, completion of questionnaires, and the wheelchair skills test. Training will last 4 -7 days. Inter- and intra- rater reliability will be calculated to determine that data being collected is consistent across collectors and sites. Should data collectors not be consistent after first training session, additional training sessions will be conducted using the protocol from IRR-03-003-1 (HSR&D current funded study on wheelchair falls in SCI).

J. Data Collection Protocol

(1) <u>*Baseline data collection*</u> will occur either in subjects' homes or at the VA and will include two performance tests: *Wheelchair Skills Test* and *Smart Wheel.* This will take approximately 1.5 hours. The subject will be given surveys to complete, which can be done on site or taken home and mailed back [Health Related Survey (Education pre-test), CHART, Attitude scale, Zuckerman Sensation Seeking Scale, CES-D, Pain and Spasticity VAS, and CHIEF]. The subject will receive a datalogger to attach to the wheelchair for 2 weeks, and will be asked to return this in a pre-paid envelope. Subjects randomized to the WSTP will be given the practice subject log that they are required to complete each day that documents amount of time spent practicing. Routine phone contacts with patients will be conducted to remind subjects about returning surveys, datalogger, and logs; this is also a subject retention strategy useful in longitudinal studies.

(2) <u>*Immediate Post-Intervention data collection*</u> is identical to baseline with the following exceptions: (a) the Zuckerman Sensation Seeking Scale will be omitted; (b) the Demographic Data Form/chart review will be omitted; (c) the completed subject logs will be collected. (d) a Health Related Survey (Education post-test) will be administered,

(3) <u>*One-Year Follow Up data collection*</u> is identical to post intervention. At conclusion the subject will be thanked for their participation.

K. Human Subject Issues: This study was approved by the local R&D Committee (May 2006). The study will be approved for adherence to Human Subjects Protection by the Institutional Review Board (Just in time approval) at all three sites. Subjects will be issued a coded identifier, which will be known only to the PI, data manager, and clerk; all records and the informed consent documents will be retained in locked files in the data manager's office. All computer data will be encrypted to protect patient confidentiality. HIPAA regulations will be strictly adhered to, as discussed previously. Data will be secure on a networked computer system behind two firewalls, and access will be restricted and password protected.

L. Women and Minorities: Our study will use a convenience sample, obtained through patient recruitment posters. Per VA policy, we will include women and minorities in this study to the extent that they are present at the two participating sites. We will make every effort to promote participation of women and minorities, such as designing study promotional products that are culturally and gender sensitive. Approximately 5% of the potential subjects are female, and approximately 23% are minorities, with comparable numbers of African Americans, and a slightly higher population of Hispanics in Tampa and Asians in Boston.

M. Multi-site Protocol: Tampa VA will act as the coordinating center for this multi-site study. Augusta and Boston will participate in subject recruitment and data collection. Every week, the clinical coordinators from Augusta and Boston will send the original consents and completed data collection forms to Tampa. This data will be sent via a traceable mail form such as USP,

Fed-Ex or Express mail. The project manager will review all forms to ensure they are complete, and will contact data collectors for additional information, if necessary. Data will be collected on scannable forms, and scanned using *Teleform* software and a high-speed printer. *Teleform* scanning allows the data to be directly placed into a database for subsequent data cleaning and analysis using SAS. We have used *Teleform* for four years in a variety of projects and have found it to be accurate, reliable, and efficient. Data will be housed in Tampa, and will be kept in a locked file cabinet in a research building that is kept locked with an entrance manned by security personnel. Due to HIPAA and Institutional Review Board (IRB) guidelines, any personal identifying information transmitted from the patient unit to the research office needs to remain private and confidential. These guidelines allow these data to be either faxed, electronically transmitted in an encrypted manner or mailed via traceable and secure carrier such as FedEx, UPS, or USPS to the Data Manager in Tampa. This system has been used in several previous studies, and it ensures confidentiality and adheres to all HIPAA guidelines. Conference calls will be scheduled regularly for investigators and research staff. Since the team has worked together in previous studies, we have developed effective and efficient communication strategies.

N. Analysis Plan: Descriptive statistics will be reported for the two groups. Chi square and t-tests will be used to compare the baseline characteristics of the groups and to test the effectiveness of the randomization. Those variables found to be statistically significant will be adjusted for in the multivariate analysis. The unit of analysis is study subject. All primary outcome measures for this study are on a continuous scale. All quantitative analysis will be performed by using Statistical Analysis System (SAS 9.1) software.

(1) Analysis for H1, H2, and Q1: As a preliminary analysis, group differences with respect to various outcomes at time 1 (immediate post-intervention) and time 2 (1-year post-intervention) will be analyzed by using simple linear regression analysis. The dependent variable will be one of the outcomes and the independent variable will be the group, a categorical variable with two levels. Separate analysis will be done for the ability, performance time and physical strain outcomes. Mixed regression analysis approach will be used to study the effect of intervention at time 1 and time 2 in one model. This approach is chosen over alternatives, e.g. repeated measures analysis of variance, mainly because it makes use of all available data when some of the repeated-measures data are missing, while repeated measure analysis of variance techniques generally require complete data. This approach will allow us to take into account the covariance pattern of the repeated measurements[205]. Ignoring the covariance issue will result in incorrect conclusions. Since the question of interest is whether the time effects differ among the two groups, time-group interaction effect will be included in the model. The model will be adjusted for the demographic (e.g. age), mediating (e.g. attitudes, self efficacy) and moderating variables (e.g., spasticity, environmental factors, extent of wheelchair). A bivariate analysis will be done to study the association of various variables with the outcomes. Those variables found to be significant at alpha 0.10 or hypothesized to be important will be included in the model and tested. Independent variables may be continuous, such as age, or categorical, such as gender. Dummy coding will be done for the categorical independent variables.

(2) Analysis for H3: For CHART, raw scores are transformed to a scale score ranging from 0 to 100 in each scale, where a score of 100 represents no handicap, a value reached by almost all able-bodied, working age adults; a score of 0 denotes complete handicap. Subscale scores will be added together to obtain a CHART total score, reflecting overall handicap level. High subscale and total scores (100 and 500, respectively) indicate less handicap, or higher social and community participation. SWLS will be used to assess the QOL. The SWLS is a 5-item global measure of life satisfaction. Scores range from 5 to 35, with higher scores representing

greater life satisfaction. Mixed regression analysis approach as outlined above will be used. For CHART, analysis will be done on total score as well as separately for each domain.

(3) *Analysis for H4*: To study the group differences with respect to the safety scores, same mixed models regression approach as outlined above will be used.

O. Missing Data: Persistent efforts will be made to obtain the highest possible degree of data completeness. Nevertheless, despite best efforts, there will be a certain fraction of subjects in whom the data will be missing. Multiple methods are available for replacing the missing data and based on the extent and randomness of the missing data, appropriate methods will be used. To the extent that missing data are completely random and small a complete case approach will be adopted, otherwise imputation or model-based approach will be used. The PROC MIXED procedure is particularly appropriate for use in longitudinal studies where dropouts are not uncommon, because the statistical theory on which PROC MIXED is based allows for unbalanced and missing data, as long as the data are missing at random.[206]

P. Quality of Data. The validity of the conclusions reached depends partly on the accuracy of the data. Every effort will be made to identify and correct errors in data collection, coding, and data entry. Interviewers for the baseline data will be trained to ensure that consistent methods of data collection are used within the interviewer as well as across sites of data collection (training plan for therapist/therapy aide have been described previously). All data forms will be reviewed prior to the subject leaving, to ensure that no questions are left blank. A checklist will be incorporated into the subject folder that will ensure that all data is collected. Interviewers will be trained to complete these forms for each participant. The Project Manager or Data Manager will review all subject folders after data collection to ensure that all data has been collected. This will be done prior to entry into the *Teleform* system. Interviewers will also be observed on a regular basis to ensure that they are collecting data in a consistent manner. Data cleaning will begin during data collection. This will allow us to detect and correct any systematic errors in data collection, coding, or entry before an enormous amount of data is collected. Measures of frequency, central tendency and dispersion, and minimum and maximum values will be used to identify errors.[207] If any error found, it will be corrected in the raw data. Date on which the data were recorded and by whom it was recorded will be noted. Care will be taken to ensure that sufficient backup procedures are in place. To assure intact data files, we will copy the raw data file into a data file for computation and analysis so that if there are computational errors or computer problems, the raw data will remain unchanged.

Q. Dissemination of Findings: We will publish findings in peer-reviewed journals and present findings at national meetings, such as VA HSR&D Annual Meetings (Nelson & Powell-Cope), American Academy of Physical Rehabilitation (Sabharwal), American Paraplegia Society (Harrow and Nelson), Rehabilitation Engineering and Assistive Technology Society of North America (RESNA) (Fitzgerald), Association of Rehabilitation Nurses (Nelson), and others. Findings will be exported throughout the VHA through the National Center for Patient Safety, the Spinal Cord Injury Strategic Health Group, and SCI QUERI. We expect several products to emerge from this study: (1) Evidence-based recommendations for incorporation of wheelchair skills testing and training as part of the life-long SCI continuum of care offered by the VA; (2) Wheelchair skills training protocol which includes the content, process, and expected outcomes; the protocol will be patient-centered (using qualitative data obtained from patients) as well as practical; (3) Cost report addressing estimated marginal costs of adding wheelchair skills

testing and testing to the SCI annual evaluation and the SCI system of care, providing an economic basis for policy and practice change. (4) Innovative marketing plans to increase patient participation and promote patient-centeredness based on patient-perceived benefits, (5) Hypothesis and research questions, grounded in the qualitative data, for further study.

V. PROJECT MANAGEMENT PLAN

TABLE 3: PROGRAM ACTIVITIES, RESPONSIBILITY, AND TIME FRAMES

Program Activities	Person(s) Responsible	Duration	Study Month
Start up activities: Recruit study personnel. Refine study brochure.	Kip, Nelson, Sabharwal, Mitchell	3 months	1-3
Construction of the obstacle courses	Applegarth, Kirby, Groer	5 months	1-5
Customize training modules for therapists and for patients to meet VA needs	Kirby, Nelson, Mitchell, Lanning, McGovern	5 months	1-5
Convert data collection instruments to Teleform	Kip, Groer	5 months	1-5
Train data entry clerk	Groer, Kip	1 month	5
Train data collectors	Kirby, Groer	1 month	6
Establish reliability of data collectors	Groer, Kirby	1 month	6
Set up data base	Kip supervised by Groer	1 month	6
Recruit, screen, and consent patients/ collect baseline data	Data Collectors under supervision of Groer & Ardila, with support from Sabharwal (Boston site) and Harrow (Tampa site), Mitchell (Augusta site)	10 months	6-15
Data Collection Wave 1		13 months	7-20
Data Collection Wave 2		13 months	10-22
Data Collection Wave 3		13 months	13-25
Data Collection Wave 4		13 months	16-28
Data entry	Byrd supervised by Ardila and Groer	24 months	6-29
Data Cleaning, Validity Checks	Ardila, McCranie, supervised by Groer		
Data Analysis	Ardila, Groer, Nelson	5 months	29-33
Final Report, Manuscripts	All investigators, led by Nelson and Groer	4 months	33-36

A. Facilities and Resources: The study will be conducted at three VA SCI Centers (Tampa, Augusta and Boston). Letters of support from the Hospital Directors are attached. Recruitment of subjects will occur in SCI inpatient and outpatient settings. Data will be managed at the Tampa VA. *Access to Patients*: Of the 1051 Veterans currently enrolled in the Tampa SCI registry, approximately 196 meet inclusion criteria (i.e., use a manual wheelchair, injury T1 or below, and live within 100 miles of the James A. Haley VA Hospital). Veterans currently enrolled in the Boston SCI registry, approximately 162 meet inclusion criteria. Since we need to recruit 170 subjects, this represents 47% of SCI patient population available at the three sites. Of the total 358 eligible SCI veterans, approximately 5% are female, and approximately 23% are minorities. *Access to Data Processing Capacity*: We have the necessary access, technology, and trained staff to effectively process data for this study. The Tampa VA Patient Safety Center of Inquiry has built significant infrastructure to support data processing capacity. We have nine staff trained in SAS, three experienced data managers, a programmer, and five staff trained to extract data from VA databases. Data will be collected on scannable forms, and scanned using *Teleform* software and a high-speed printer. *Teleform* scanning allows the data to be directly placed into a database for subsequent data cleaning and analysis using SAS. We have used *Teleform* for 7 years in a variety of projects and have found it to be accurate, reliable, and efficient. Data will be managed by Mark McCranie. He will supervise the data entry/automation

clerk, train data collectors, develop and pilot test the *Teleform* data entry system, validate 10% of data entry, clean data, and perform preliminary data analyses. He will be supervised by Dr. Groer, an epidemiologist at the Tampa VA. *Access to Space:* Most key research staff already have offices at the Tampa VA, and space will be provided for the data collectors and Project Manager. The office will include a workstation and access to phone.

B. Role and Tasks of Research Team. Each investigator is briefly described, including discipline, role and responsibilities in the study, accomplishments, and expertise. This description is outlined in the Budget Justification pages. **Collaboration:** With a 3 year track record of collaboration on anther HSR&D study [Epidemiology and Cost of Falls in Veterans with a Spinal Cord Injury [IRR-03-003-1], this team will continue to communicate electronically as well as by conference calls, and at national meetings where many of the investigators will be attending (e.g., annual American Paraplegia Society Meeting). Additionally, we have scheduled several meetings outlined in Budget Justification.

REFERENCES

[1] Jones, M., & Sanford, J. A. (1996). People with mobility impairments in the United States today and in 2010. *Assist Technol, 8,* 43-53.
[2] Pierce, L. L. (1998). Barriers to access: frustrations of people who use a wheelchair for full-time mobility. *Rehabilitation Nursing,* 23(3), 120-125.
[3] Kirby, R.L., Ackroyd-Stolarz, S.A., Brown, M., Kirkland, S. A., & MacLeod, D.A. (1994). Ibid.
[4] Silfverskiold, J., Waters, R. L. (1991). Shoulder pain and functional disability in spinal cord injury patients. *Clinical Orthopaedics & Related Research, Nov*(272), 141-145.
[5] van Drongelen, S., de Groot, S., Veeger, H. E., Angenot, E. L., Dallmeijer, A. J., Post, M. W. et al. (2006). Ibid.
[6] Waring, W. P., & Maynard, F. M. (1991). Shoulder pain in acute traumatic quadriplegia. *Paraplegia, 29*(1), 37-42.
[7] Sie, I. H., Waters, R. L., Adkins, R. H., & Gellman, H. (1992). Upper extremity pain in the post rehabilitation spinal cord injured patient. *Arch Phys Med Rehabil, 73,* 44-48.
[8] Pentland, W. E., Twomey, L. T. (1991). The weight-bearing upper extremity in women with long term paraplegia. *Paraplegia, 29,*521-530.
[9] Gellman, H., Sie, I., & Waters, R. L. (1988). Late complications of the weight-bearing upper extremity in the paraplegic patient. *Clinical Orthopaedics & Related Research, 233*, 132-135.
[10] Sie, I. H., Waters, R. L., Adkins, R. H., & Gellman, H. (1992). Ibid.
[11] Pentland, W. E., Twomey, L. T. (1991). Ibid.
[12] Gellman, H., Sie, I., & Waters, R. L. (1988). Ibid.
[13] Bayley, J. C., Cochran, T. P., & Sledge, C.B. (1987). The weight-bearing shoulder. The impingement syndrome in paraplegics. *J Bone Joint Surg [Am], 69,* 676-678.
[14] Wylie, E. J., & Chakera, T. M. (1988). Degenerative joint abnormalities in patients with paraplegia of duration greater than 20 years. *Paraplegia, 26,*101-106.
[15] Gellman, H., Chandler, D. R., Petrasek, J., Sie, I., Adkins, R., & Waters, R.L. (1988). Carpal tunnel syndrome in paraplegic patients. *J Bone Joint Surg [Am], 70,* 517-519.
[16] Sie, I. H., Waters, R. L., Adkins, R. H., & Gellman, H. (1992). Ibid.
[17] Burnham, R. S., & Steadward, R. D. (1994). Upper extremity peripheral nerve entrapments among wheelchair athletes Prevalence, location, and risk factors. *Arch Phys Med Rehabil, 75,* 519-524.
[18] Tun, C. G., & Upton, J. (1988). The paraplegic hand: Electrodiagnostic studies and clinical findings. *J Hand Surg [Am], 13,* 716-719.
[19] Aljure, J., Eltorai, I., Bradley, W. E., Lin, J. E., & Johnson, B. (1985). Carpal tunnel syndrome in paraplegic patients. *Paraplegia, 23,* 182-186.
[20] Davidoff, G., Werner, R., & Waring, W. (1991). Compressive mononeuropathies of the upper extremity in chronic paraplegia. *Paraplegia, 29,* 17-24.
[21] Silfverskiold, J., Waters, R. L. (1991). Ibid.
[22] Curtis, K. A., Roach, K. E., Applegate, E. B., Amar, T., Benbow, C. S., Genecco, T. D. et al. (1995). Development of the Wheelchair User's Shoulder Pain Index (WUSPI). *Paraplegia, 33*(5), 290-293.
[23] Pentland, W. E., & Twomey, L. T. (1994). Upper limb function in persons with long term paraplegia and implications for independence: Part II. *Paraplegia, 32*(4), 219-224.
[24] Calder, C. J., & Kirby, R. L. (1990). Fatal wheelchair-related accidents in the United States. *Amer J Phys Med Rehabil, 69*, 184-190.
[25] Preservation of Upper Limb Function Following a Spinal Cord Injury: A Clinical Practice Guideline for Health-Care Professionals. (2004). Retrieved April 17, 2005, from http://www.pva.org/publications/pdf/upperlimb.pdf.
[26] Gray, D. B., Hollingsworth, H. H., Stark, S. L., & Morgan, K. A. (2006). Participation survey/mobility: psychometric properties of a measure of participation for people with mobility impairments and limitations. *Arch Phys Med Rehabil, 87,* 189-197.
[27] Xiang, H., Chany, A. M., & Smith, G. A. (2006). Wheelchair related injuries treated in US emergency departments. *Inj Prev, 12*(1), 8-11.
[28] Ummat, S., & Kirby, R. L. (1994). Nonfatal wheelchair-related accidents reported to the National Electronic Injury Surveillance System. *Am J Phys Med Rehabil, 73*, 163-167.
[29] Xiang, H., Chany, A. M., & Smith, G. A. (2006). Ibid.

[30] Ummat, S., & Kirby, R. L. (1994). Ibid.
[31] Ummat, S., & Kirby, R. L. (1994). Ibid.
[32] Whiteneck, G., Meade, M. A., Dijkers, M., Tate, D. G., Bushnik, T., & Forchheimer, M. B. (2004). Environmental factors and their role in participation and life satisfaction after spinal cord injury. *Arch Phys Med Rehabil, 85*(11), 1793-1803.
[33] Gaal, R. P., Rebholtz, N., Hotchkiss, R. D., & Pfaelzer, P. F. (1997). Wheelchair rider injuries: causes and consequences for wheelchair design and selection (p. 59). *Journal of Rehabilitation Research & Development, 34*(1), 58-71.
[34] Kirby, R.L., Ackroyd-Stolarz, S.A., Brown, M., Kirkland, S. A., & MacLeod, D.A. (1994). Ibid.
[35] Calder, C. J., Kirby, R. L. (1990). Ibid.
[36] Kirby, R. L., & Ackroyd-Stolarz, S. A. (1995). Wheelchair safety: adverse reports to the United States Food and Drug Administration. *Amer J Phys Med Rehabil, 74,* 308-312.
[37] Yogi,T. (2000). Information for wheelchair Users. Available at: http://www.members.cruzio.com/~yogi/whchair.htm [12-13-00].
[38] Nelson, A. L., Ahmed, S., Harrow, J., Fitzgerald, S., Sanchez-Anguiano, A. & Gavin-Dreschnack, D. (2003). Fall-related fractures in persons with spinal cord impairment: a descriptive analysis. *SCI Nursing, 20* (1), 30-37.
[39] Nelson, AL, Markee, S., Sanchez-Anguiano, A., Gavin-Dreschnack, D., Fitzgerald, S., Harrow, J, Powell-Cope, G., Quigley, P., Ahmed, S., & French, D. (In Review). Fractures Associated with Falls in Veterans with SCI. *Spinal Cord Nursing.*
[40] Calder, C. J., Kirby, R. L. (1990). Ibid.
[41] Kirby, R.L., Ackroyd-Stolarz, S.A., Brown, M., Kirkland, S. A., & MacLeod, D.A. (1994). Ibid.
[42] Gaal, R. P., Rebholtz, N., Hotchkiss, R. D., & Pfaelzer, P. F. (1997). Ibid.
[43] Calder, C. J., Kirby, R. L. (1990). Ibid.
[44] Kirby, R.L., Ackroyd-Stolarz, S.A., Brown, M., Kirkland, S. A., & MacLeod, D.A. (1994). Ibid.
[45] Nelson, AL, Ahmed, S., Harrow, J, Fitzgerald, S., Sanchez-Anguiano, A. and Gavin-Dreschnack, D., (2003). Ibid.
[46] Kirby, R.L., Ackroyd-Stolarz, S.A., Brown, M., Kirkland, S. A., & MacLeod, D.A. (1994). Ibid.
[47] Product Summary Report of the National Electronic Injury Surveillance System. (1987). Washington, DC: US Consumer Product Safety Commission National Injury Information Clearinghouse.
[48] Kirby, R.L., Ackroyd-Stolarz, S.A., Brown, M., Kirkland, S. A., & MacLeod, D.A. (1994). Ibid.
[49] Berg, K., Hines, M., & Allen, S. (2002). Wheelchair users at home: few home modifications and many injurious falls. *American Journal of Public Health, 92*(1), 48.
[50] Nichols, P. J., Norman, P. A., & Ennis, J. R. (1979). Wheelchair user's shoulder? Shoulder pain in patients with spinal cord lesions. *Scand J Rehabil Med, 11*, 29-32.
[51] Gellman, H., Sie, I., & Waters, R. L. (1988). Ibid.
[52] Sie, I. H., Waters, R. L., Adkins, R. H., & Gellman, H. (1992). Ibid.
[53] Pentland, W. E., & Twomey, L. T. (1994). Ibid.
[54] Wylie, E. J., & Chakera, T. M. (1988). Ibid.
[55] Lal, S. (1998). Premature degenerative shoulder changes in spinal cord injury patients. *Spinal Cord, 36*(3), 186-189.
[56] Sie, I. H., Waters, R. L., Adkins, R. H., & Gellman, H. (1992). Ibid.
[57] Gellman, H., Chandler, D. R., Petrasek, J., Sie, I., Adkins, R., & Waters, R. L. (1988). Ibid.
[58] Tun, C. G., & Upton, J. (1988). Ibid.
[59] Burnham, R. S., & Steadward, R. D. (1994). Ibid.
[60] Preserving Upper Limb Function in Spinal Cord Injury: A Clinical Practice Guideline for Health-Care Professionals. 2004. Consortium for Spinal Cord Medicine. Spinal Cord Medicine, Clinical Practice Guideline.
[61] Bonaparte, L. P., Kirby, R. L. , MacLeod, D. A. (2004). Learning to perform wheelchair wheelies: comparison of 2 training strategies. *Archives of Physical Medicine and Rehabilitation, 85,* 785-93.
[62] MacPhee, A. , H. , Kirby, R. L., Coolen, A. L., Smith, C., MacLeod, D. A. , Dupuis, D. J. (2004). Wheelchair skills training program: a randomized clinical trial on wheelchair users undergoing initial rehabilitation. *Archives of Physical Medicine & Rehabilitation, 85,* 41-50.
[63] Gray, D. B., Hollingsworth, H. H., Stark, S. L., & Morgan, K. A. (2006). Ibid.
[64] MacPhee, A. H., Kirby, R. L., Coolen, A. L., Smith, C., MacLeod, D. A., Dupuis, D. J. (2004). Ibid.

65 Gray, D. B., Hollingsworth, H. H., Stark, S. L., & Morgan, K. A. (2006). Ibid.
66 Gaal, R. P., Rebholtz, N., Hotchkiss, R. D., & Pfaelzer, P. F. (1997). Ibid.
67 Loane, T. D., & Kirby, R. L. (1985) Static rear stability of conventional and lightweight variable-axle-position wheelchairs. *Arch Phys Med Rehabilitation, 66*, 174-176.
68 Kirby, R. L., MacLean, A. D., & Eastwood, B.J. (1992). Influence of caster diameter on the static and dynamic forward stability of occupied wheelchairs. *Arch Phys Med Rehabil, 73*, 73-77.
69 Gaal, R. P., Rebholtz, N., Hotchkiss, R. D., & Pfaelzer, P. F. (1997). Ibid.
70 Trudel, G., Kirby, R. & Bell, A. (1995). Mechanical effects of rear-wheel camber on wheelchairs. *Assistive Technology, 7*, 79-86.
71 Trudel, G., Kirby, R. L., Ackroyd-Stolarz, S. A., & Kirkland, S. (1997). Effects of rear-wheel camber on wheelchair stability. *Archives of Physical Medicine and Rehabilitation, 78*, 78-81.
72 Kirby, R.L., Ackroyd-Stolarz, S.A., Brown, M., Kirkland, S. A., & MacLeod, D.A. (1994). Ibid.
73 Kirby, R. L., Ashton, B. D., Ackroyd-Stolarz, S. A., & MacLeod, D. A. (1996). Adding loads to occupied wheelchairs: effect on static rear and forward stability. *Arch Phys Med Rehabil, 77*, 183-166.
74 Coolen, A. L., Kirby, R. L., Landry, J., MacPhee, A. H., Dupuis, D., Smith, C., Best, K. L., Mackenzie, D. E., & MacLeod, D. A. (2004) Wheelchair skills training program for clinicians: a randomized controlled trial with occupational therapy students. *Arch Phys Med Rehabil. 85*(7), 1160-1167.
75 MacPhee, A. H., Kirby, R. L., Coolen, A. L., Smith, C., MacLeod, D. A., Dupuis, D. J. (2004). Ibid.
76 Kilkens, O. J., Dallmeijer, A. J., Nene, A. V., Post, M. W., & van der Woude, L. H. (2005). The longitudinal relation between physical capacity and wheelchair skill performance during inpatient rehabilitation of people with spinal cord injury. *Arch Phys Med Rehabil,* 86(8), 1575-1581.
77 Kilkens, O. J., Post, M. W., Dallmaijer, A. J., Seelen, H. A., & van der Woude, L. H. (2003). Wheelchair skills tests: a systematic review. *Clinical Rehabilitation,* 17(4), 418-430.
78 Bullard, S., & Miller, S. E. (2001). Comparison of teaching methods to learn a tilt and balance wheelchair skill. *Precept Mot Skills, 2001, 93*(1), 131-138.
79 Kirby, R. L., Mifflen, N. J., Thibault, D. L., Smith, C., Best, K. L., Thompson, K. J., & MacLeod, D. A. (2004) The manual wheelchair-handling skills of caregivers and the effect of training. *Arch Phys Med Rehabil, 85*, 2011-2019.
80 Coolen, A. L., Kirby, R. L., Landry, J., MacPhee, A. H., Dupuis, D., Smith, C., Best, K. L., Mackenzie, D. E., & MacLeod, D. A. (2004). Ibid.
81 Best, K. L., Kirby, R. L., Smith, C., MacLeod, D. A. (2005). Wheelchair skills training for community-based manual wheelchair users: a randomized controlled trial. *Arch Phys Med Rehabil, 86*(12), 2316-2323.
82 MacPhee, A. H., Kirby, R. L., Coolen, A. L., Smith, C., MacLeod, D. A., Dupuis, D. J. (2004). Ibid.
83 Kirby, R. L., Mifflen, N. J., Thibault, D. L., Smith, C., Best, K. L., Thompson, K. J., & MacLeod, D. A. (2004). Ibid.
84 Kirby, R. L., Smith, C., Seaman, R., MacLeod, D. A., & Parker, K. (2006). The manual wheelchair wheelie: a review of our current understanding of an important motor skill. *Disability & Rehabilitation: Assistive Technology, 1,* 119-127.
85 Paralyzed Veterans of America. (2005). Clinical Practice Guidelines: Preservation of Upper Extremity Function Following SCI. Retrieved April 17, 2006, from http://www.pva.org/res/cpg/devgde.htm.
86 Paralyzed Veterans of America. (2005). Preservation of Upper Extremity Function Following SCI. Clinical Practice Guidelines. Retrieved April 17, 2006, from http://www.pva.org/res/cpg/devgde.htm.
87 Kirby, R. L., Smith, C., Seaman, R., Macleod, D. A., Parker, K.. (2006). Ibid.
88 Kirby, R. L.., Swuste, J., Dupuis, D. J., MacLeod, D. A., & Monroe, R. (2002). The wheelchair skills test: a pilot study of a new outcome measure. *Arch Phys Med Rehabil, 83*(1), 10-18.
89 Dalhousie University Faculty of Medicine (2006). *The Wheelchair Skills Training Program*. Retrieved April 18, 2006, from www.wheelchairskillsprogram.ca.
90 MacPhee, A. H., Kirby, R. L., Coolen, A. L., Smith, C., MacLeod, D. A., Dupuis, D. J. (2004). Ibid.
91 Best, K. L., Kirby, R. L., Smith, C., MacLeod, D. A. (2005). Ibid.
92 Coolen, A. L., Kirby, R. L., Landry, J., MacPhee, A. H., Dupuis, D., Smith, C., Best, K. L., Mackenzie, D. E., & MacLeod, D. A. (2004). Ibid.
93 Kilkens, O. J., Dallmeijer, A. J., Nene, A. V., Post, M. W., & van der Woude, L. H. (2005). Ibid.
94 Kilkens, O. J., Post, M. W., Dallmeijer, A. J., van Asbeck, F. W., & van der Woude, L. H. (2005) Relationship between manual wheelchair skill performance and participation of persons with spinal cord

injuries 1 year after discharge from inpatient rehabilitation. *J Rehabil Res Dev, 42*(3 Suppl 1), 65-73.

95 Kilkens, O. J., Post, M. W., Dallmeijer, A. J., van Asbeck, F. W., & can der Woude, L. H. (2005) Ibid.

96 National Center for Health Statistics (NCHS). (2005). International Classification of Diseases and Functioning and Disability. Retrieved April 17, 2006, from http://www.cdc.gov/nchs/about/otheract/icd9/icfhome.htm.

97 Putnam, M. (2002), Linking aging theory and disability models: increasing the potential to explore aging with physical impairment. *Gerontologist, 42*(6), 799-806.

98 Putnam M. (2002), Ibid.

99 Brandt, E. N., & Pope, A. M. (1997). *Enabling America: Assessing the role of Rehabilitation Engineering*. Institute of Medicine, Washington DC: National Academies Press.

100 O'Donnell, M.P. (1989). Definition of Health Promotion: Part III: Expanding the definition. *American Journal of Health Promotion, 3*(3), 5.

101 Patrick, D. L. (1997). Rethinking prevention for people with disabilities. Part I: A conceptual model for promoting health. *American Journal of Health Promotion, 11*, 257-260.

102 Patrick, D. L. (1997). Ibid.

103 WHO. International Classification of Functioning, Disability and Health (ICF). Geneva: World Health Organization, 2001.

104 Kilkens, O. J., Post, M. W., Dallmeijer, A. J., van Asbeck, F. W., & can der Woude, L. H. (2005). Ibid.

105 Brechtelsbauer , D.A. & Louie, A. (1999). Wheelchair use among long-term care residents. *Annals of Long Term Care, 7*(6), 213-220.

106 http://www.members.cruzio.com/~yogi/whchair.htm [12-13-00].

107 Kilkens, O. J., Post, M. W., Dallmeijer, A. J., van Asbeck, F. W., & can der Woude, L. H. (2005). Ibid.

108 Kilkens, O. J. , Post, M. V., Dallmeijer, A. J., Seelen, H. A. ,van der Woude, L. H. (2003). Ibid.

109 Institute of Medicine (2002). *Speaking of Health: Assessing health communication strategies for diverse populations.* Washington, DC: National Academies Press, pg. 29.

110 Bandura A. (1986). *Social Foundations of Thought and Action: A Social Cognitive Theory*. Englewood Cliffs, NJ: Prentice Hall.

111 Montano, D., & Kasprzyk, D. (2002). The theory of reasoned action and the theory of planned behavior. In K. Glanz, B. Rimer, & F. Lewis (Eds). *Health behavior and health education: Theory, Research, and Practice, 3rd Ed*., (pp. 67-98). San Francisco: Jossey-Bass.

112 Prochaska, J., Redding, C., & Evers, K. (2002). The transtheoretical model and stages of change. In K. Glanz, B. Rimer, & F. Lewis (Eds). *Health behavior and health education: Theory, Research, and Practice, 3rd Ed*., (pp. 99-120). San Francisco: Jossey-Bass.

113 Institute of Medicine (2002). Ibid.

114 Friedman, Y., Hart-Hughes, S., Quigley, P., & Spehar, A.M. (In press). Fall prevention clinic: deployment and initial outcome measures. *Federal Practitioner.*

115 Neily, J., Howard, K., Quigley, P., & Mills, P. D. (2005). One-year follow-up after a collaborative breakthrough series on reducing falls and fall-related injuries. *The Joint Commission Journal on Quality and Safety, 31*(5), 275-285.

116 Quigley, P., Bulat, T., & Hart-Hughes, S. (In press). Evidence-based strategies to reduce risk of fall-related injuries. *Journal of Rehabilitation Nursing.*

117 Quigley, P., Palacios, P., & Spehar, A. (In press). Fall risk: VISN-wide fall risk prevalence study. *Clinical Interventions in Aging.*

118 Mills, P. D., Waldron, J., Quigley, P., Stalhandske, E., & Weeks, W. B. (2005). Case Study: Reducing Falls and Injuries Due to Falls in the VA System (pp. 117-145). In: *The JCAHO 2005 National Patient Safety Goals: Successful Strategies for Compliance*. Oakbrook Terrace, IL: JCAHO.

119 Quigley, P. (2005). Falls research agenda (Guest Editorial). *Journal of Rehabilitation Research and Development, 42*(1), vii-x.

120 Nelson, A. L., Ahmed, S., Harrow, J., Fitzgerald, S., Sanchez-Anguiano, A. & Gavin-Dreschnack, D. (2003). Ibid.

121 Gavin-Dreschnack, D., Nelson, A., Fitzgerald, S., Harrow, J., Sanchez-Anguiano, A., Ahmed, S., & Powell-Cope, G. (2005). Wheelchair-related falls: current evidence and directions for improved quality care. *J Nurs Care Qual.,20*(2), 119-127.

[122] MacPhee, A. H., Kirby, R. L., Coolen, A. L., Smith, C., MacLeod, D. A., Dupuis, D. J. (2004). Ibid.
[123] Best, K. L., Kirby, R. L., Smith, C., & MacLeod, D. A. (2005). Ibid.
[124] Coolen, A. L., Kirby, R. L., Landry, J., MacPhee, A. H., Dupuis, D., Smith, C., Best, K. L., Mackenzie, D. E., & MacLeod, D. A. (2004). Ibid.
[125] Kirby, R. L., Mifflen, N. J., Thibault, D. L., Smith, C., Best, K. L., Thompson, K. J., & MacLeod, D. A. (2004). Ibid.
[126] U.S. Census. (2000). www.census2000
[127] Kirby, R.L. & Ackroyd-Stolarz, S.A. (1995). Ibid.
[128] Kirby, R.L., Ackroyd-Stolarz, S.A., Brown, M., Kirkland, S. A., & MacLeod, D.A. (1994). Ibid.
[129] Silfverskiold, J., Waters, R. L. (1991). Ibid.
[130] van Drongelen, S., de Groot, S., Veeger, H. E., Angenot, E. L., Dallmeijer, A. J., Post, M. W. et al. (2006). Ibid.
[131] Sie, I. H., Waters, R. L., Adkins, R. H., & Gellman, H. (1992). Ibid.
[132] Pentland, W. E., Twomey, L. T. (1991). Ibid.
[133] Gellman, H., Chandler, D. R., Petrasek, J., Sie, I., Adkins, R., & Waters, R.L. (1988). Ibid.
[134] Sie, I. H., Waters, R. L., Adkins, R. H., & Gellman, H. (1992). Ibid.
[135] Calder, C. J., & Kirby, R. L. (1990). Ibid.
[136] Department of Veterans Affairs (VHA). (2005). VHA Handbook 1176.1: Spinal Cord Injuries and Disorders System of Care Procedures. Retrieved April 17, 2005, from http://www1.va.gov/vhapublications/ViewPublication.asp?pub__ID=1255.
[137] Campbell, R. R., Bradham, D. D., Sanchez-Anguiano, A., Werner, D. C., Quigley, P., & Spehar, A. M. (2003). Developing the VHA Falls Injury Surveillance System (FISS). White paper. Tampa, FL: HSR&D REAP.
[138] Campbell, R. R., Bradham, D. D., Sanchez-Anguiano, A., Werner, D. C., Quigley, P., & Spehar, A. M. (2003). Ibid.
[139] Department of Veterans Affairs (VHA). (2005). Ibid.
[140] Choi, H., Binder, D.S., Oropilla, M.L., Bernotus, E.E., Konya, D., Nee, M.A., Tammaro, E.A., Sabharwal, S. (2006).Evaluation of Selected Laboratory Components of a Comprehensive Periodic Health Evaluation for Veterans With Spinal Cord Injury and Disorders. *Arch Phys Med, 87*(5), 603-610.
[141] Dick, M. B., Hseih, S., Bricker, J., Dick-Muehlke, C. (2003). Facilitating acquisition and transfer of a continous motor task in healthy older adults and patients with Alzheimer's Disease. *Neuropsychology, 17,* 202-212.
[142] McNevin, N. H. , Wulf, G., Carlson, C. (2000). Effects on attentional focus, self-control, and dyad training on motorlearning: implications for physical rehabilitation. *Physical Therapy, 80,* 373-385.
[143] Wulf, G., HöB, M., Prinz, W. (1998). Instructions for motor learning: differential effects of internal versus external focus of attention. *J Mot Behav, 30*, 169-179.
[144] Magill, R. A., & Schonfelder-Zohdi, B. (1996). A visual model and knowledge of performance as source of information for learning a rhythmis gymnastic skill. *Int J Sport Psychol, 27*, 7-22.
[145] Weeks, D. L., & Kordus, R. N. (1998). Relative frequency of knowledge of performance and motor skill learning. *Res Q Exerc Sport, 69,* 224-230.
[146] The Wheelchair Skills Program. Manual can be downloaded from: www.wheelchairskillsprogram.ca
[147] Cohen, J. (1988). Statistical power analysis for the behavioral sciences. (2nd ed.) Hillsdale, NJ: Lawrence Erlbaum Associations.
[148] MacPhee, A. H., Kirby, R. L., Coolen, A. L., Smith, C., MacLeod, D. A., Dupuis, D. J. (2004). Ibid.
[149] Best KL, Kirby RL, Smith C, MacLeod DA. (2005). Ibid.
[150] Coolen AL., Kirby RL, Landry J, MacPhee AH, Dupuis D, Smith C, Best, KL, MacKenzie DE, MacLeod DA. (2004). Ibid.
[151] Kilkens, O. J., Dallmeijer, A. J., Nene, A. V., Post, M. W., van der Woude, L. H. (2005). Ibid.
[152] Kilkens, O. J., Post, M. W., Dallmeijer, A. J., van Asbeck, F. W., van der Woude, L. H. (2005). Ibid.
[153] Hintze, J. L. (2000). PASS 2000 user's guide. Kaysville, UT: Number Cruncher Statistical Systems.
[154] Lipsitz, S. R., Fitzmaurice, G. M., Sleeper, L., & Zhao, L. P. (1995). Estimation methods for the join distribution of repeated binary observations. *Biometrics, 51*(2), 562-570.

[155] Vickers, A. J. (2003). How many repeated measures in repeated measures designs? Statistical issues for comparative trials. *BMC Med Res Methodol, 3*(1), 22.
[156] Freidman, LM, Furberg, CD & DeMets, DL (1998). *Fundamentals of Clinical Trials*. NY: Springer-Verlag
[157] Freidman, LM, Furberg, CD & DeMets, DL (1998). Ibid.
[158] Freidman, LM, Furberg, CD & DeMets, DL (1998). Ibid.
[159] Freidman, LM, Furberg, CD & DeMets, DL (1998). Ibid.
[160] Kirby, R. L., Swuste, J., Dupuis, D. J., MacLeod, D. A. & Monroe, R. (2002). Ibid.
[161] Kirby R. L., Dupuis, D. J., MacPhee, A. H., Coolen, A. L., Smith, C., Best, K. L., Newton, A. M., Mountain, A. D., MacLeod, D. A., & Bonaparte, J. P. (2004). The Wheelchair Skills Test (version 2.4): measurement properties. *Arch Phys Med Rehabil, 85*, 794-804.
[162] Kilkens, O. J., Post, M. W., Dallmeijer, A. J., Seelen, H. A., & van der Woude, L. H. (2003). Ibid.
[163] Best, K. L., Kirby, R. L., Smith, C., & MacLeod, D. A. (2005). Ibid.
[164] Best, K. L., Kirby, R. L., Smith, C., & MacLeod, D. A. (2006). Comparison between performance with a pushrim-activated power-assisted wheelchair and a manual wheelchair on the Wheelchair Skills Test. *Disabil Rehabil, 28*(4), 213-220.
[165] MacPhee, A. H., Kirby, R. L., Coolen, A. L., Smith, C., MacLeod, D. A., Dupuis, D. J. (2004). Ibid.
[166] Coolen, A. L., Kirby, R. L., Landry, J., MacPhee, A. H., Dupuis, D., Smith, C., Best, K. L., Mackenzie, D. E., & MacLeod, D. A. (2004) Ibid.
[167] Bonaparte, J. P., Kirby, R. L., & Macleod, D. A. (2004). Ibid.
[168] Cooper, R. A., Robertson, R. N., VanSickle, D. P., Boninger, M. L., & Shimada, S. D. (1997). Methods for determining three-dimensional wheelchair pushrim forces and moments: a technical note. *J Rehabil Res Dev, 34*(2), 162-170.
[169] Newsam, C L., Mulroy, S. J., Gronley, J. K., Bontrager, E. L., & Perry, J. (1996). Temporal-spatial characteristics of wheelchair propulsion. Effects of level of spinal cord injury, terrain, and propulsion rate. *Am J Phys Med Rehabil, 73*(4), 292-299.
[170] Koonz, A. M., Cooper, R. A., Boninger, M. L., Yang, Y., Impink, B. G., & van der Woude, L. H. V. (2005). A kinetic analysis of manual wheelchair propulsion during start-up on select indoor and outdoor surfaces.
[171] Boninger, M. L., Cooper, R. A., Robertson, R. N., & Shimada, S. D. (1997). 3-D Pushrim Forces During Two Speeds of Wheelchair Propulsion. Am J Phys Med Rehabil, 76(5), 420-426.
[172] Boninger, M. L., Koontz, A. M., Sisto, S. A., Dyson-Hudson, T. A., Chang, M., Price, R. et al. (2005). Pushrim biomechanics and injury prevention in spinal cord injury: Recommendations based on CULP-SCI Investigations. *J Rehab Res Dev, 42*(3, Supp. 1), 9-20.
[173] Fitzgerald, S., Arva, J., Cooper, R., Dvorznak, M., Spaeth, D., & Boninger, M. (2003). A pilot study on community usage of a pushrim-activated, power-assisted wheelchair. *Assist Technol, 15*(2), 113-119.
[174] Tolerico, M. L., Ding, D., Cooper, R. A., Spaeth, D. M., Fitzgerald, S. G., Cooper, R. et al. (In press). Assessing the mobility characteristics and levels of manual wheelchair users in two real world environments. *Journal of Rehabilitation Research and Development*.
[175] Craig Hospital. (2001). Craig Hospital Inventory of Environmental Factors, Version 3.0. Retrieved April 17, 2006, from http://www.craighospital.org/Research/Disability/DISCHIEF.asp
[176] Whiteneck, G., Meade, M. A., Dijkers, M., Tate, D. G., Bushnik, T., & Forchheimer, M. B. (2004). Ibid.
[177] Whiteneck, G. G., Harrison-Felix, C. L., Mellick, D. C., Brooks, C. A., Charlifue, S. B., & Gerhart, K. A. (2004). Quantifying environmental factors: a measure of physical, attitudinal, service, productivity, and policy barriers. *Archives of Physical Medicine & Rehabilitation, 85*(8), 1324-1335.
[178] Richards, J. S., Bombardier, C. H., Tate, D., Dijkers, M., Gordon, W., Shewchuk, R., & DeVivo, M. J. (1999). Access to the environment and life satisfaction after spinal cord injury. *Arch Phys Med Rehabil, 80*(11), 1501-1506.
[179] Whiteneck, G. G., Charlifue, S. W., Gerhart, K. A., Overholsert, J. D., & Richardson, G. N. (1992). Quantifying handicap: a newmeasure of long-term rehabilitation outcomes. *Arch Phys Med Rehabil, 73*(6), 519-526.
[180] Whiteneck, G., Tate, D., & Charlifue, S. (1999). Predicting community reintegration after spinal cord injury from demographic and injury characteristics. *Arch Phys Med Rehabil, 81*(2), 164-169.
[181] Kazis, L. (1997). *The SF-36V: Veterans Health Study*. Technical Report from the Center for Health Quality, Outcomes, & Economic Research.
[182] Ware, J., & Sherbourne, C. (1992). The MOS-36 Short-Form health Survey (SF-36). *Medical Care, 30*,

473-483.
183 Kazis LE, Miller DR, Clark J, Skinner K, Lee A, Rodgers W, Spiro A, Payne S, Fincke G, Selim A, Linzer M. (1998). Health-related quality of life in patients served by the department of veterans affairs. *Arch Intern Med, 158,* 626-632.
184 Ware JE. (2000). SF-36 health survey update. *Spine, 25,* 3130-3139.
185 Kazis LE. Miller DR. Skinner KM. Lee A. Ren XS. Clark JA. Rogers WH. Spiro A 3rd. Selim A. Linzer M. Payne SM. Mansell D. Fincke RG. (2004). Patient-reported measures of health: The Veterans Health Study, *Journal of Ambulatory Care Management. 27*(1):70-83.
186 Ajzen, I. & Fishbein, M. (1980). *Understanding attitudes and predicting social behavior*. Englewood Cliffs, NJ: Prentice-Hall.
187 Ajzen, I, & Madden, T. (1986). Prediction of goal-directed behavior: Attitudes, intentions, and perceived behavioral control. *Journal of experimental social psychology, 22,* 453-474.
188 Montano, D., Kasprzyk, D., & Taplin, S. (2002). Ibid.
189 Young, H.M., Lierman, L., Powell-Cope, Kasprzyk, D., & Benoliel, J. (1991). Operationalizing the theory of planned behavior. *Research in Nursing & Health, 14*, 137-144.
190 Blue, C.(1995). The predictive capacity of the theory of reasoned action and the theory of planned behavior in exercise research: review of the literature. *Research in Nursing & Health, 18,* 105-121.
191 Jemmott, L., & Jemmott, J. (1991). Applying the theory of reasoned action to AIDS risk behavior: condom use among black women *Nursing Research, 40*, 228-234.
192 Madden, T., Ellen, P. & Ajzen, I. (1992). A comparison of the theory of planned behavior and the theory of reasoned action. *Personality and Social Psychology Bulletin, 18*(1), 3-9.
193 Powell-Cope, G. (2001). Adherence to Antiretroviral Therapy in HIV-Infected Adults. Final report to VA HSR&D, HSR&D/NRI Proposal #97-005.
194 Luther, S., Nelson, A., Powell-Cope, G. (2004, November). Provider Attitudes and Beliefs about Clinical Practice Guidelines. *Spinal Cord Injury Nursing, 21*(4), 206-212.
195 Neugaard, B., Luther, S., Powell-Cope, G., Escabi, J., & Nelson, A. (October 2004) Measuring Provider Compliance with Ischemic Heart Disease Guidelines. *Southern Medical Journal, 97(*10), 942-947.
196 Zuckerman M, Kolin EA, Price L & Zoob I. Development of a sensation-seeking scale. J Consult Psychol 28: 477-82. 1964.
197 Roberts RE. Reliability of the CES-D Scale in different ethnic contexts. Psychiatry Res. 1980 May;2(2):125-34
198 Weissman MM, Sholomskas D, Pottenger M, Prusoff BA, Locke BZ. Assessing depressive symptoms in five psychiatric populations: a validation study. Am J Epidemiol. 1977 Sep;106(3):203-214
199 Price DD, Bush FM, Long S, Harkins SW. A comparison of pain measurement characteristics of mechanical visual analogue and simple numerical rating scales. Pain. 1994 Feb;56(2):217-26.
200 Stormer S, Gerner HJ, Gruninger W, Metzmacher K, Follinger S, Wienke C,Aldinger W, Walker N, Zimmermann M, Paeslack V. Chronic pain/dysaesthesiae in spinal cord injury patients: results of a multi centre study. Spinal Cord. 1997 Jul;35(7):446-55
201 Kirshblum S. Treatment alternatives for spinal cord injury related spasticity. J Spinal Cord Med. 1999 Fall;22(3):199-217.
202 Skold C. Spasticity in spinal cord injury: self- and clinically rated intrinsic fluctuations and intervention-induced changes. Arch Phys Med Rehabil. 2000 Feb;81(2):144-9.
203 Lechner, H. E., Frotzler, A., & Eser, P. (2006). Relationship between self- and clinically rated spasticity in spinal cord injury. *Arch Phys Med Rehabilitation 87*(1), 15-19.
204 Schulz KF, Grimes DA. Generation of allocation sequences in randomized trials: chance, not choice. Lancet. 2002 Feb 9;359(9305):515-9.
205 Brown, H. & Prescott, R. (1999). *Applied mixed models in medicine*, West Sussex, U.K.: Wiley, ISBN 0-471-96554-5
206 Littell, R. C., Milliken, G. A., Stroup, W. W., & Wolfinger, R. D. (1996) *SAS System for Mixed Models*. Cary, NC: SAS Institutes.
207 Suter, W. N. (1987). Approaches to avoiding errors in data sets. *Nursing Research, 36*, 262-263.
